# Endometrial Epithelial Lactate Deficiency Drives CD8^+^ T‐Cells Dysregulation in Unexplained Recurrent Implantation Failure

**DOI:** 10.1002/advs.202524090

**Published:** 2026-02-27

**Authors:** Yuanlin He, Kang Ke, Qingxia Meng, Yichun Guan, Jichun Tan, Hong Lv, Boxian Huang, Chan Tian, Qinyan Zou, Mingxing An, Yao Gao, Jingjing Chen, Xiaoyu Wei, Xi Wang, Hongxia Ma, Yuan Lin, Feiyang Diao, Yayun Gu, Zhibin Hu

**Affiliations:** ^1^ State Key Laboratory of Reproductive Medicine and Offspring Health Nanjing Medical University Nanjing Jiangsu China; ^2^ Innovation Center of Suzhou Nanjing Medical University Suzhou Jiangsu China; ^3^ Department of Epidemiology Center for Global Health School of Public Health Nanjing Medical University Nanjing China; ^4^ Reproductive Genetic Center The Affiliated Suzhou Hospital of Nanjing Medical University Suzhou Municipal Hospital Gusu School Nanjing Medical University Suzhou Jiangsu China; ^5^ Center for Reproductive Medicine The Third Affiliated Hospital of Zhengzhou University Zhengzhou China; ^6^ Center of Reproductive Medicine Department of Obstetrics and Gynecology Shengjing Hospital of China Medical University Shenyang China; ^7^ State Key Laboratory of Reproductive Medicine and Offspring Health (Suzhou Centre) The Affiliated Suzhou Hospital of Nanjing Medical University Suzhou Municipal Hospital Gusu School Nanjing Medical University Suzhou China; ^8^ Department of Maternal Child and Adolescent Health Center for Global Health School of Public Health Nanjing Medical University Nanjing China; ^9^ State Key Laboratory of Female Fertility Promotion Center for Reproductive Medicine Department of Obstetrics and Gynecology Peking University Third Hospital Beijing China; ^10^ Department of Immunology Nanjing Medical University Nanjing China; ^11^ Clinical Center of Reproductive Medicine The First Affiliated Hospital of Nanjing Medical University Nanjing Medical University Nanjing Jiangsu China

**Keywords:** abnormal glycolysis, CD8^+^ T‐cells, endometrial, glandular epithelium, recurrent implantation failure

## Abstract

Recurrent implantation failure (RIF), characterized by repeated failure to achieve clinical pregnancy after embryo transfer, is often associated with abnormal endometrial conditions. However, unexplained RIF is unique in that its underlying cause remains largely unknown, posing a challenge for both clinicians and patients. We performed single‐cell RNA sequencing and reported that the endometria of patients with unexplained RIF exhibited increased the proliferation and activation of cytotoxic CD8^+^ T‐cells, which hinder embryo implantation. Additionally, the glandular epithelium, which has the highest metabolic activity, showed abnormal glycolysis and reduced lactate production in RIF. Specifically, the endometrial expression of genes associated with glucose uptake (*SLC2A1*), glucose metabolism (*ALDOA*), lactate production (*LDHA*), and lactate output (*SLC16A3*) were lower in the patients with unexplained RIF than in the controls. Through uterine horn injection experiments in mice, we demonstrated that inhibiting lactate production in the endometrium prevents embryo implantation and that this effect could be reversed by lactate supplementation. Moreover, lactate inhibitors did not affect implantation in mice with CD8^+^ T‐cells depletion. In vitro experiments also confirmed that lactate inhibition affects the proliferation and activation of CD8^+^ T‐cells. We propose that the endometria of patients with unexplained RIF fail to establish proper immune balance toward the embryo, likely due to abnormal glycolysis and reduced lactate production in the glandular epithelium.

## Graphical abstract

Unexplained recurrent implantation failure (RIF) represents a significant clinical challenge. Our results demonstrate that reduced lactate production in the RIF endometrium impairs the suppression of cytotoxic CD8^+^ T‑cells, allowing their proliferation and thereby disrupting the local immune balance essential for successful embryo implantation. This metabolic alteration directly compromises the endometrial immunological milieu, ultimately leading to implantation failure.

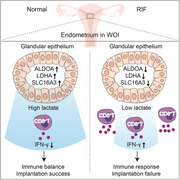

## 1 Introduction

Assisted reproductive technology (ART) has significantly increased fertility, yet many couples struggle to conceive even after multiple treatment cycles [1]. Recurrent implantation failure (RIF) is a major clinical challenge, but its definition is inconsistent across studies. A common definition is failure to achieve a clinical pregnancy after the transfer of at least 4 good‐quality embryos in a minimum of three fresh or frozen cycles in a woman under the age of 40 years [2]. The newest consensus added that an RIF diagnosis should focus on failure to achieve “sustained” implantation, which is defined as a gestational sac identified on ultrasound, allowing differentiation from recurrent pregnancy loss [3]. The causes of RIF include embryo abnormality, parental chromosomal abnormalities, endocrine and metabolic diseases, autoimmune diseases, and endometrial diseases such as endometrial structural diseases, endometritis, and endometriosis [2]. Approximately two‐thirds of implantation failures have been reported to be attributable to factors related to the endometrium [4]. However, unexplained RIF represents a particularly challenging subgroup that lacks distinct pathological characteristics and presents more challenges in treatment [5].

Pregnancy involves complex processes, including implantation, decidualization, placentation, and parturition [6]. Implantation, the initial stage of pregnancy, occurs when the embryo adheres to and invades within the endometrium [7]. Optimal endometrial conditions are crucial for successful implantation. Inadequate endometrial receptivity (ER), defined as the capacity of the endometrium to accept and accommodate embryos, is a primary cause of implantation failure [4].

The human menstrual cycle is primarily divided into two phases: proliferative and secretory [8]. Throughout the menstrual cycle, the endometrium undergoes dynamic structural and biochemical changes [9]. During the mid‐secretory phase, the endometrium reaches an optimal state for embryo implantation known as the “window of implantation” (WOI) [10]. At this time, the endometrial immune system transitions to a state of immune tolerance, preparing to accept the semi‐allograft embryo [11]. Consequently, immune activation within the endometrium impedes embryo implantation, as observed in various implantation‐related diseases such as endometritis [12]. Previous studies have shown that natural killer (NK) cells progressively increase following successful embryo implantation and support fetal growth [13, 14]. However, the roles of other immune cells in implantation and the regulatory mechanisms governing these cells during the WOI remain unclear. Similarly, metabolic regulation plays an important role in uterine receptivity during early pregnancy, ultimately influencing pregnancy efficiency in mammals [15]. However, the mechanisms underlying metabolism during early pregnancy are not yet fully understood, and the interplay between metabolism and the immune response warrants further investigation.

In the present study, we used single‐cell RNA sequencing and mouse models to investigate the endometrial mechanism underlying unexplained RIF. Our findings indicate that, compared with controls, the endometria of patients with unexplained RIF have more immune cells, especially CD8^+^ T‐cells, which secrete interferon gamma (IFNG). Furthermore, the glandular epithelium in the endometria of RIF patients exhibits abnormal glycolysis and insufficient lactate production, which impairs immune tolerance, resulting in a greater proportion of endometrial toxic CD8^+^ T‐cells and thereby RIF. These insights offer a new perspective on the diagnosis and treatment of unexplained RIF.

## 2 Results

### 2.1 Cohort of Patients With Unexplained RIF

**FIGURE 1:**
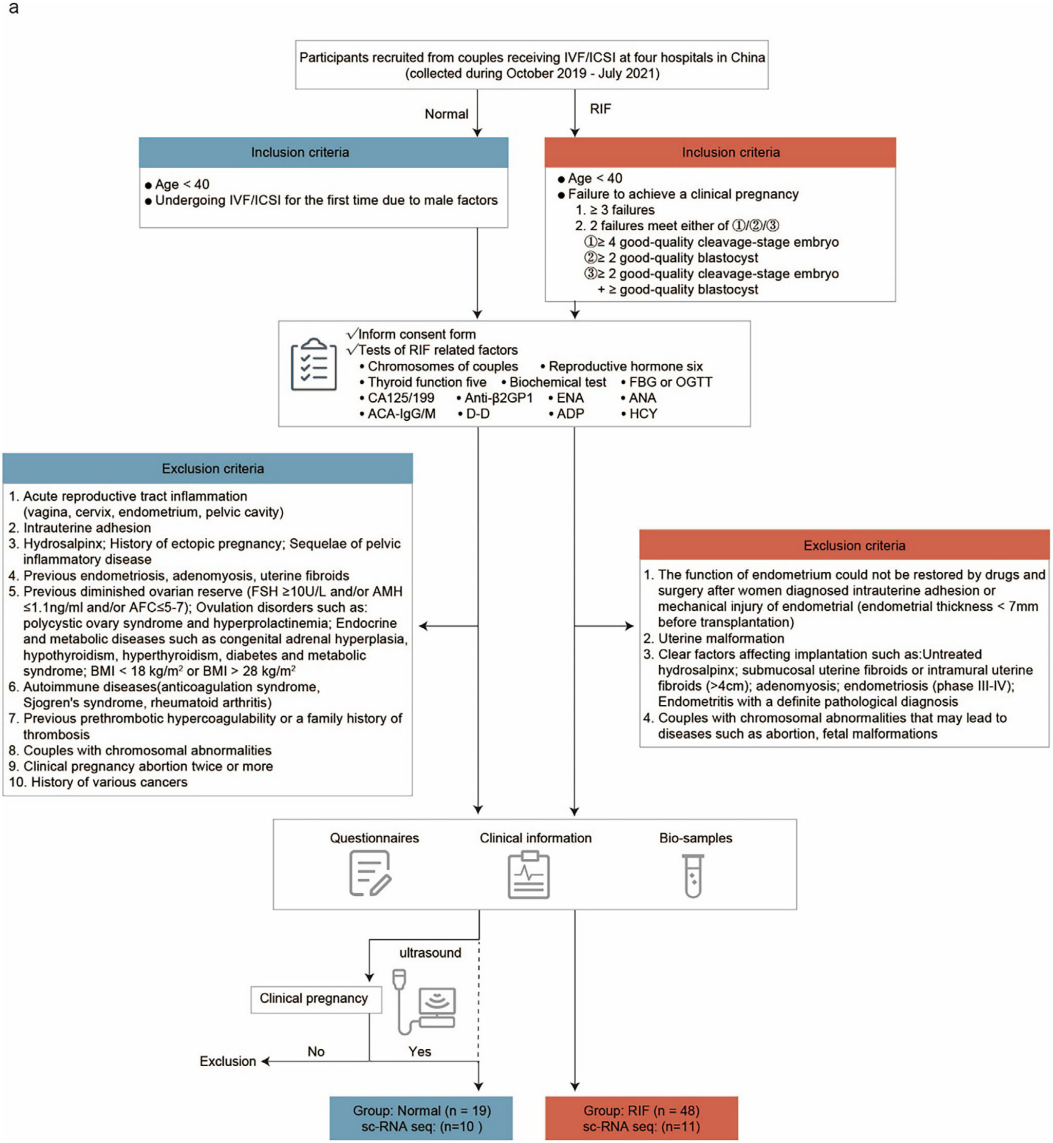
Cohort of patients with unexplained RIF. (a) Patients meeting the inclusion criteria were recruited for the initial assessment, during which medical records and serum tests were assessed for each participant. After candidates with specific exclusion criteria were excluded, the remaining participants completed questionnaires and underwent clinical data collection and sample collection. In addition, candidates in the control group were required to have had previously confirmed clinical pregnancy. All questionnaires and sample collection were conducted by trained physicians by prescribed procedures. (b) Candidates in the control group were required to confirm a clinical pregnancy by ultrasound before they are finally identified as a part of control group.

To investigate the risk factors for and provide etiological evidence of unexplained RIF, we established a cohort of patients with RIF by recruiting participants from among couples receiving IVF/ICSI. Eligible participants included women under 40 years of age who had repeatedly failed to achieve a clinical pregnancy. The control group consisted of women who underwent ART for the first time due to male factor infertility and successfully achieved a clinical pregnancy. Finally, 48 patients with unexplained RIF and 19 controls were included, and endometrial biopsies from 11 patients with unexplained RIF and 10 controls were used for single‐cell RNA sequencing (scRNA‐seq) (Figure 1a). There were no significant differences between the patients with unexplained RIF and the controls in terms of age, body mass index, hormone endocrine function, liver function, kidney function, or other metabolic parameters. In addition, the endometrial thickness was within the normal range in both groups, suggesting that samples were collected during the appropriate phase for implantation (Table S1).

### 2.2 Single‐Cell Transcriptome Profiling Revealed Deficient Immune Balance in the Endometria of Patients With Unexplained RIF

**FIGURE 2:**
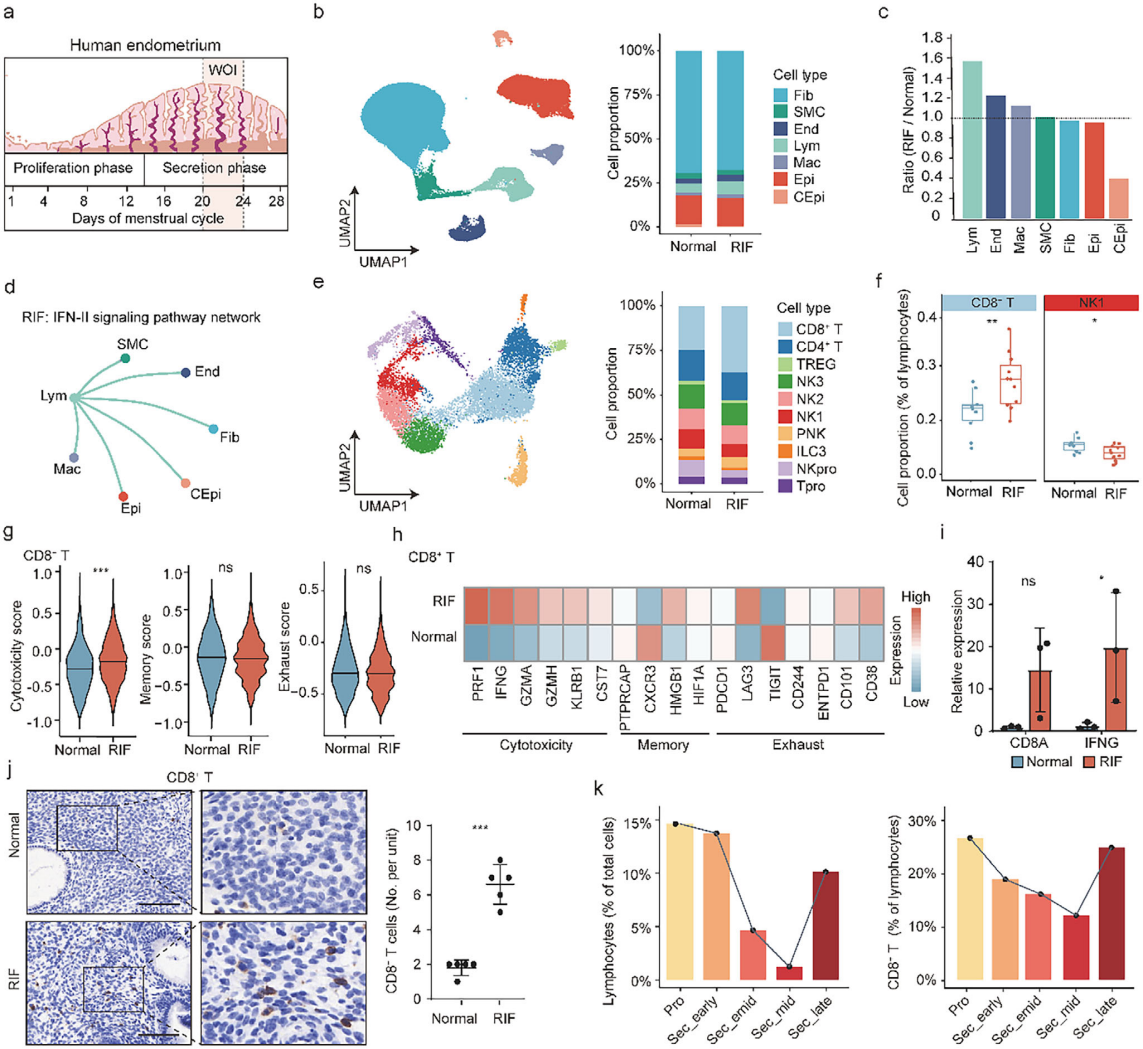
Single‐cell transcriptome profiling reveals deficient immune balance in the endometria of patients with unexplained RIF. (a) Schematic diagram of the endometrium during the menstrual cycle. Biopsy samples were collected during the WOI (10 controls and 11 patients with unexplained RIF). (b) Left: UMAP projections of all sequenced cells from 10 controls and 11 patients with unexplained RIF; seven major cell types were identified. Fib: Fibroblasts, SMC: Smooth muscle cells, End: Endothelial cells, Lym: Lymphocyte, Mac: Macrophage, Epi: epithelial cells, CEpi: Ciliated epithelial cells. Right: The proportions of the seven major cell types in the endometria of the controls and patients with unexplained RIF. (c) Bar plot showing the differences in the proportions of seven major cell types between the endometria of the controls and patients with unexplained RIF. (d) Circle plot showing the interaction weight networks from lymphocytes to other cells in the patients with unexplained RIF. (e) Left: UMAP plot showing the ten subsets of lymphocytes. Right: The proportions of the lymphocyte subsets in the endometria of the controls and patients with unexplained RIF. (f) The proportions of CD8^+^ T cell and dNK1 cell subsets among the lymphocytes from the controls and patients with unexplained RIF. Data are presented as median with IQR. The lower and upper hinges correspond to the first and third quartiles (the 25th and 75th percentiles). The upper (lower) whisker extends from the hinge to the largest (smallest) value no further than 1.5 * IQR from the hinge. *p* values were calculated by two‐sided unpaired Mann–Whitney *U* test (Normal, n  =  10; RIF, n  =  11). *, *p* < 0.05; **, *p* < 0.01. (g) Gene set enrichment analysis of the indicated signatures of CD8^+^ T cell subsets. *p* values were calculated by two‐tailed Student's *t*‐test. ***, *p* < 0.001. (h) Heatmap showing the relative gene expression of the indicated signatures of CD8^+^ T cell subsets from the controls and patients with unexplained RIF. (i) The differences in the mRNA expression levels of *CD8A* and *IFNG* between the endometria from the controls and patients with unexplained RIF were examined by RT‒qPCR (n = 3 per group). The data are presented as the mean ± s.d. and were analyzed by two‐tailed Student's *t*‐test. *, *P* < 0.05. (j) Left: Immunohistochemical staining for the expression of CD8A in the endometria from the controls and patients with unexplained RIF. Scale bar, 100 µm. Right: The number of CD8^+^ T‐cells per field in endometria from the controls and patients with unexplained RIF. The data are presented as the mean ± s.d. and were analyzed by two‐tailed Student's *t*‐test. ***, *p* < 0.001. (k) Changes in the proportions of lymphocytes (left) and CD8^+^ T–cells (right) among lymphocytes from the endometrium throughout the menstrual cycle. Data included endometrial samples from 10 controls across different phases of the menstrual cycle.

In the scRNA‐seq data, a total of 188 198 cells were captured during the WOI from the endometrial tissues of 11 patients with unexplained RIF and 10 controls (Figure 2a). After quality control and the removal of doublets, 93 782 cells from patients with unexplained RIF and 86 075 cells from the controls were used for further analysis. Dimensional reduction via RunUMAP on combined cells from both groups identified seven distinct cell groups, as reported previously (Figure 2b and Figure S1a–e) [16]. Among these seven cell types, those that make up the endometrium proper, namely, stromal fibroblasts (Fib), epithelial cells (Epi), macrophages (Mac), endothelial cells (End), and smooth muscle cells (SMC), did not significantly differ in population size between the patients with unexplained RIF and the controls. However, the lymphocyte (Lym) population increased by nearly 50% in the patients with unexplained RIF, whereas the population of ciliated epithelial cells (CEpi) decreased by approximately 60% (Figure 2c; Figure S1f,g). To investigate the potential impact of increased lymphocytes on the abundance of other cell types, we examined the cellular communication among the seven cell types via the CellChat algorithm. The results revealed that signaling pathways related to the immune response, such as IFN‐II, IL16, COMPLEMENT, and LIFR, were predominant in the patients with unexplained RIF. In contrast, pathways related to endometrial receptivity, such as EGF, TWEAK, and CSF, were more prominent in the controls (Figure 2d; Figure S1h). Within the IFN‐II signaling pathway, the interaction pair IFNG‐IFNGR was derived from lymphocytes and targeted other cell types. Notably, the expression of *IFNG* in lymphocytes was higher in the patients with unexplained RIF than in the controls (Figure S1i,j).

Given the critical role of cellular immunity in the semi‐allograft rejection of embryos, the finding of an enlarged lymphocyte population in patients with unexplained RIF suggests that persistent immune surveillance may contribute to RIF. Further analysis of lymphocytes revealed ten subpopulations: CD8^+^ T‐cells, CD4^+^ T‐cells, regulatory T cells (Treg), three types of NK cells (NK1, NK2, and NK3), peripheral NK cells, ILC3s, proliferating NK cells and proliferating T cells (Figure 2e, Figure S2a, and Tables S2 and S3). Among these subpopulations, CD8^+^ T‐cells composed the largest population, followed by CD4^+^ T‐cells and NK cells (Figure 2e; Figure S2b). Notably, the proportion of CD8^+^ T‐cells among lymphocyte cells was significantly greater in the patients with unexplained RIF than in the controls, whereas the proportion of NK1 cells was lower (Figure 2f; Figure S2c,d). In line with classic cytotoxic CD8^+^ T‐cells functions, endometrial CD8^+^ T‐cells were characterized by higher expression of effector genes, such as *NKG7*, *GNLY*, *GZMA*, and *GZMK*. Conversely, immune regulated genes, including *TNF*, *KLRB1*, and *IL7R* were highly expressed in endometrial CD4^+^ T‐cells (Figure S2e). Further analysis revealed that CD8^+^ T‐cells presented the greatest number of differentially expressed genes among all the lymphocyte cells (Figure S2f). CD8^+^ T‐cells exhibited higher expression of cytotoxic genes, but not of genes related to memory or exhaustion, in the patients with unexplained RIF than in the controls (Figure 2g,h; Figure S2g).

To further elucidate the effect of low levels of CD8^+^ T‐cells on implantation, we employed CIBERSORTx to estimate the relative frequencies of CD8^+^ T‐cells in endometrial samples based on RNA sequencing data. Consistent with the results of scRNA‐seq, this analysis revealed a greater proportion of CD8^+^ T‐cells in the endometria of patients with unexplained RIF than in those of the controls (Figure S3a). Real‐time PCR verified increased expression of *CD8A* and *IFNG* in the endometria of patients with unexplained RIF (Figure 2i). Immunohistochemistry (IHC) of human endometrial tissue revealed more CD8^+^ T‐cells but fewer CD4^+^ T‐cells and CD56^+^ cells in the patients with unexplained RIF compared with the controls (Figure 2j; Figure S3b). Additionally, we utilized an external dataset to determine whether the proportion of CD8^+^ T‐cells decreased during the WOI compared with other phases of the menstrual cycle [16]. This dataset included endometrial samples from 10 controls across different phases of the menstrual cycle, including the proliferative phase and four secretory phases (early, early‐middle, middle, and late) (Figure S3c). The proportions of both lymphocytes and CD8^+^ T‐cells decreased from the proliferative phase to the secretory phase and reached a minimum during the mid‐secretory phase (i.e., the WOI) (Figure 2k).

### 2.3 The Glandular Epithelia of Patients With Unexplained RIF Exhibit Immature Differentiation

**FIGURE 3:**
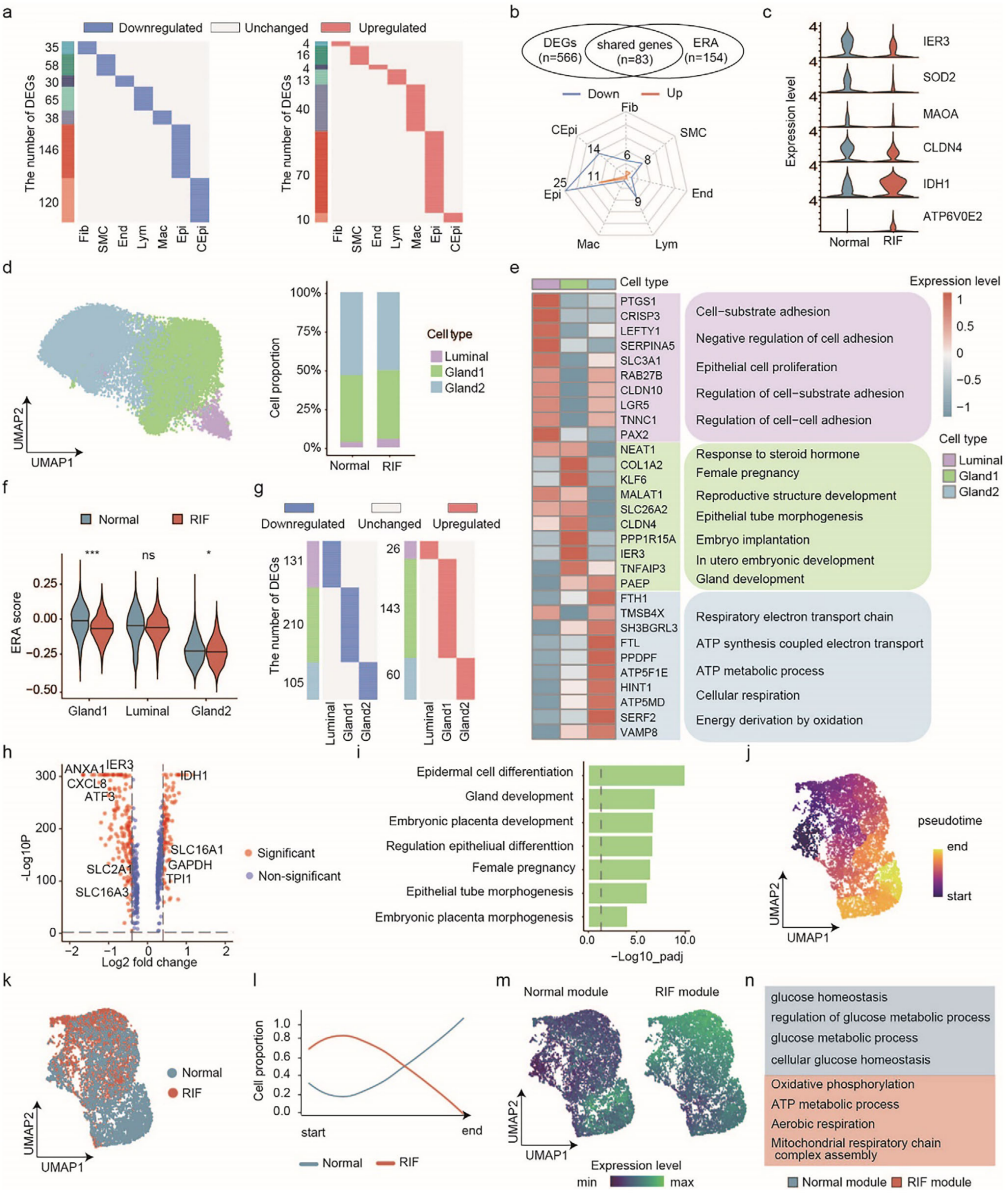
Glandular epithelia of patients with unexplained RIF exhibited immature differentiation. (a) Heatmaps showing the distribution of DEGs between the patients with unexplained RIF and the controls in each endometrial cell type. (b) Upper: Venn diagrams showing the overlaps (83 shared genes) between DEGs and the ERA. Lower: Radar chart showing the number of shared genes in the seven cell types. (c) Violin plot showing the expression of partially shared genes between DEGs and the ERA in the epithelia of the controls and patients with unexplained RIF. (d) Left: UMAP plot showing three subsets of epithelial cells. Right: The proportions of epithelial subsets in the controls and patients with unexplained RIF. (e) Left: Heatmap showing the expression signatures of the top 10 specifically expressed genes in each epithelial subset; the value for each gene is the row‐scaled Z score. Right: representative Gene Ontology (GO) terms. (f) Gene set enrichment analysis for the ERA signature of three epithelial subsets in the controls and patients with unexplained RIF. The data are presented as the mean ± s.d. and were analyzed by two‐tailed Student's *t*‐test. *, *p* < 0.05; ***, *p* < 0.001. (g) Heatmaps showing the distribution of DEGs between the patients with unexplained RIF and the controls in the three epithelial subsets. (h) Volcano plots representing differentially expressed genes within the glandular epithelium in the controls and patients with unexplained RIF. (i) GO analysis of the DEGs in the glandular epithelium between the controls and patients with unexplained RIF. (j) UMAP dimensionality reduction in the glandular epithelia from the controls and patients with unexplained RIF. Each cell is colored according to its pseudotime cell trajectory assignment. (k) UMAP visualization of the glandular epithelia from the controls and patients with unexplained RIF. (l) The relative proportion of glandular epithelia in the controls and patients with unexplained RIF along the pseudotime cell trajectory. (m) UMAP of module scores for gene signatures in the controls and patients with unexplained RIF (see Supplementary Note for a complete list of genes). (n) Representative GO terms of the module genes in the glandular epithelia of the controls and patients with unexplained RIF.

We next sought to identify RIF‐associated genes expressed in endometrial cells. Differential expression analysis revealed 492 downregulated and 157 upregulated differentially expressed genes (DEGs) in the patients with unexplained RIF compared with the controls (Figure 3a, Figure S4a, and Table S4). Among the 7 cell types, those that form the unciliated epithelium presented the greatest number of differentially expressed genes, regardless of whether the genes were upregulated or downregulated, suggesting that this expression pattern in patients with unexplained RIF is abnormal. The Endometrial Receptivity Array (ERA) gene set was then used to measure endometrial receptivity [17], and the ERA scores were the highest during the WOI (Figure S4b) and in cells of the unciliated epithelium (Figure S4c). We further counted the number of DEGs shared between of the analyses of different cell types and the ERA. Among the 83 shared genes, 25 that were downregulated and 11 that were upregulated were associated with cells of the unciliated epithelium, representing the highest count among all cell types (Figure 3b,c; Figure S4d).

We next focused on the unciliated epithelium and further identified three subsets: gland1 epithelium, gland2 epithelium, and luminal epithelium (Figure 3d; Figure S4e,f). No significant differences were observed in the proportions of these three subsets between the patients with unexplained RIF and the controls (Figure 3d; Figure S4g). Gene Ontology (GO) characteristics related to “respiratory electron transport chain” and “ATP metabolic process” were detected across the gland2 subset, whereas the luminal subset was enriched in “cell junction organization” and “regulation of cell‐cell adhesion.” Importantly, the gland1 subset was enriched in “reproductive structure development,” “embryo implantation,” and “gland development,” indicating its crucial role in embryo implantation (Figure 3e). Furthermore, compared with the controls, the patients with unexplained RIF presented a lower ERA score for the gland1 epithelium, which had the highest score among all three subsets (Figure 3f). DEG analysis and GO analysis were further performed on the three epithelial subsets. Specifically, 105, 210, and 131 downregulated DEGs and 60, 143, and 26 upregulated DEGs (comparing patients with unexplained RIF with the controls) were identified in the gland1, gland2, and luminal subsets, respectively (Figure 3g). GO analysis revealed “negative regulation of ATP metabolic process” alterations in the gland2 subset (Figure S4h) and “regulation of cell‐substrate adhesion” alterations in the luminal subset (Figure S4i). In parallel, the gland1 subset presented downregulated expression of *ANXA1*, *SLC16A4*, *ATF3* and *SLC2A1*, which was associated with “epidermal cell differentiation” and “gland development” (Figure 3h,i).

Given the downregulated expression of differentiation‐related genes in the gland1 epithelium of patients with unexplained RIF, we utilized Monocle 3 to construct a pseudotime cell trajectory for cells in this subset (Figure 3j). The trajectory revealed progression of development from origin to maturity (Figure 3j). Notably, the patients with unexplained RIF presented a greater relative proportion of gland1 epithelia in the early stage of the trajectory, while the controls presented more gland1 epithelia in the late stage, suggesting delayed development in the gland1 epithelia in patients with unexplained RIF (Figure 3k,l, Figure S4j, and Table S5). Genes associated with the pseudotime cell trajectory were classified into coregulated modules representing distinct functions. We compared the differentially expressed modules (DEMs) between the controls and the patients with unexplained RIF to characterize their different functions along the pseudotime cell trajectory (Figure S4k and Table S6). The module for patients with unexplained RIF module was characterized by oxidative phosphorylation and ATP metabolism, whereas the module for the controls was characterized by glucose homeostasis and glucose metabolism (Figure 3m,n). These findings imply that there is aberrant energy metabolism in the gland1 epithelia of patients with unexplained RIF.

### 2.4 The Glandular Epithelia of Patients With Unexplained RIF Exhibit Abnormal Glycolysis and Reduced Lactate Production

**FIGURE 4:**
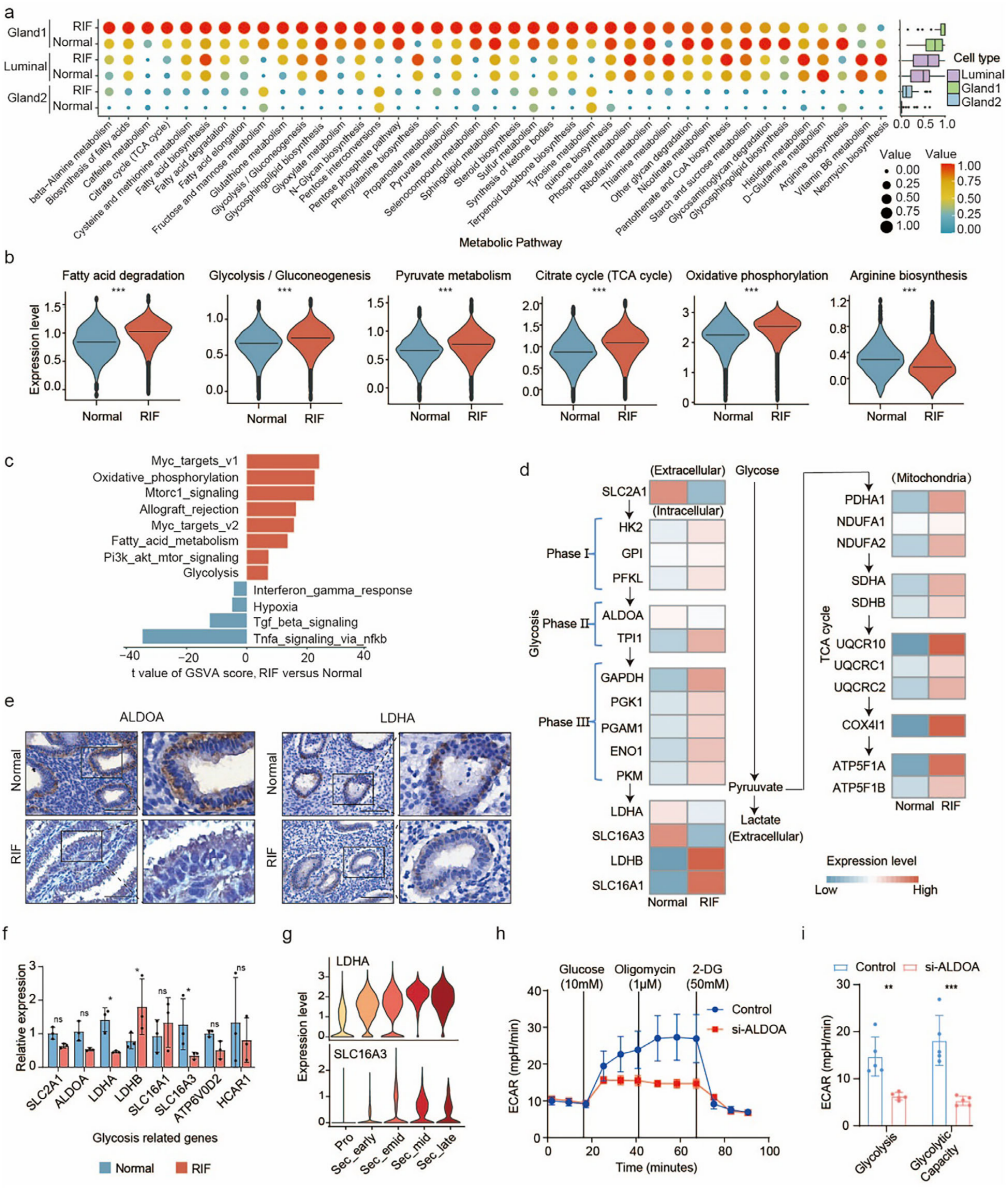
Glandular epithelia of patients with unexplained RIF exhibited abnormal glycolysis and reduced lactate production. (a) The metabolic activity analysis of the epithelium revealed that the glandular epithelium, especially that in patients with unexplained RIF, had the highest metabolic score. The circle size and colour brightness both represent the scaled metabolic score. (b) Gene set enrichment analysis for the indicated signature of the glandular epithelium. The data are presented as the mean ± s.d. and were analyzed by two‐tailed Student's *t*‐test. ***, *p* < 0.001. (c) Differences in pathway activities scored per cell by GSVA in the glandular epithelia between the controls and patients with unexplained RIF. The t values from a linear model are shown. (d) Schematic diagram of glycolysis and mitochondrial OXPHOS in the glandular epithelium. The mRNA expression of glycolysis‐related and mitochondrial OXPHOS‐related genes in the glandular epithelia of the controls and patients with unexplained RIF. (e) IHC analysis of the expression of ALDOA and LDHA in human endometrial tissues from the controls and patients with unexplained RIF. Scale bar, 100 µm. (f) The differences in the mRNA expression levels of glycolysis‐related genes and transcription factors between the endometria of the controls and patients with unexplained RIF examined by RT‐qPCR (n = 3 per group). The data are presented as the mean ± s.d. and were analyzed by two‐tailed Student's *t*‐test. *, *p* < 0.05. (g) Dynamics of LDHA and SLC16A3 expression in the glandular epithelium of normal endometria throughout the menstrual cycle. (h,i) ECAR changes, glycolysis rates, and glycolysis capacities were measured. *p* values were calculated by two‐tailed Student's *t*‐test. **, *p* < 0.01; ***, *p* < 0.001.

To investigate metabolic activity in the endometrium, we used scMetabolism to evaluate the relative metabolic activity within different cell types. Among all cell types in the endometrium, the cells of the unciliated epithelium exhibited the highest metabolic activity, indicating their highly energetic state (Figure S5a–c). Furthermore, cells of the gland1 epithelia presented the highest metabolic activity among the three epithelial subsets, and the average metabolic score of patients with unexplained RIF was greater than that of the controls (Figure 4a). Pathways related to energy metabolism, including fatty acid degradation, glycolysis, pyruvate metabolism, the citrate cycle and oxidative phosphorylation, were more enriched in the gland1 epithelia of patients with unexplained RIF, while arginine biosynthesis was more enriched in the gland1 epithelia of the controls (Figure 4b). We also identified differential hallmark pathways in the gland1 epithelia between patients with unexplained RIF and the controls via GSVA. Similarly, the results revealed that patients with unexplained RIF had greater enrichment in the energy‐associated pathways “oxidative phosphorylation,” “fatty acid metabolism,” and “glycolysis” (Figure 4c). Pathways related to glycolysis regulation, such as “MYC,” “MTORC1,” and “PI3K_AKT,” were also enriched in the gland1 epithelia of patients with unexplained RIF. Interestingly, a direct comparison of gland1 epithelia between patients with unexplained RIF versus controls revealed “allograft rejection” targets as the differentially enriched signatures. However, the gland1 epithelia of the controls were enriched in “hypoxia” and pathways related to immune tolerance (that is, pathways associated with the IFNG response, TGFβ, and TNFA signalling via NF‐kB) (Figure 4c).

We next compared the expression of glycometabolism‐related genes between patients with unexplained RIF and the controls (Figure 4d). Most genes involved in glycolysis and OXPHOS had greater expression in the gland1 epithelia of patients with unexplained RIF than in those of the controls. However, the gland1 epithelia of patients with unexplained RIF had lower expression of *SLC2A1*, a key rate‐limiting factor for glucose uptake. Furthermore, ALDOA, a key enzyme of the glycolysis pathway that accelerates glycolysis and produces lactate, was expressed at lower levels in the gland1 epithelia of patients with unexplained RIF. Considering the greater enrichment of “hypoxia” in the gland1 epithelia of the controls, we hypothesized that the controls better utilized glucose for lactate production. We then evaluated the expression of lactate‐related genes in the gland1 epithelium. The key genes *LDHA* and *SLC16A3*, which promote lactate production and secretion, were more highly expressed in the normal gland1 epithelium, whereas genes promoting lactate uptake (*SLC16A1*) and the production of pyruvate enzymes from oxidation (*LDHB*) were more highly expressed in the patients with unexplained RIF (Figure 4d). These results indicate that pyruvate, produced by glycolysis, was mostly converted into lactate in the controls but was involved in oxidative phosphorylation in patients with unexplained RIF.

We next verified the expression of lactate‐related genes and proteins in the endometrium. IHC revealed that ALDOA and LDHA localized mainly on the gland1 epithelium and were expressed at higher levels in the controls than in patients with unexplained RIF (Figure 4e and Figure S5d–f). Real‐time quantitative polymerase chain reaction (RT‒qPCR) further revealed that the expression of *SLC2A1*, *ALDOA*, *LDHA*, *SLC16A3*, and *HIF1A* was greater in the gland1 epithelia of the controls, while the expression of *LDHB* and *SLC16A1* was greater in the gland1 epithelia of patients with unexplained RIF (Figure 4f). Then, we used an external dataset to examine the expression of lactate‐related genes during different phases of the menstrual cycle. The *SLC2A1* gene affecting glucose uptake was expressed mainly in the epithelium, indicating active glycolysis (Figure S6a). In the gland1 epithelium, *SLC2A1*, *ALDOA*, *LDHA*, and *SLC16A4* were noticeably upregulated in the secretory phase compared with the proliferative phase and reached the highest levels during the WOI (Figure 4g; Figure S6b). These finding were further verified in the mice model (Figure S6c,d). Moreover, we verified the effects of *ALDOA* on glycolysis and lactate production in vitro. The extracellular acidification rate (ECAR) was used to evaluate the rate of glycolysis and the glycolytic capacity (Figure 4h;  Figure S6c, S6e). Small interfering RNA (siRNA)‐mediated *ALDOA* knockdown reduced the ECAR in ECC‐1 cells, an established epithelial cell line derived from an adenocarcinoma of the human endometrium (Figure 4h). si‐ALDOA ECC‐1 cells presented considerably decreased glycolytic rates and glycolytic capacity compared to control ECC‐1 cells (Figure 4i). Conversely, the ECAR was increased in ECC‐1 cells overexpressing *A* *LDOA* (Figure S6d–g). The overexpression of *ALDOA* increased the rate of glycolysis and the glycolytic capacity in ECC‐1 cells (Figure S6f, S6h). These in vitro results show that decreased *ALDOA* expression in patients with unexplained RIF hinders glycolysis and reduces lactate production. To elucidate the underlying cause of the aberrant glycolytic metabolism and diminished lactate production, we examined the transcriptional response of gland1 epithelial cells to ovarian hormones using publicly available endometrial organoid datasets. In this study, exogenous estrogen and progesterone were applied to simulate different phases of the menstrual cycle [18]. The results revealed that progesterone stimulation obviously upregulated the expression of genes involved in glycolysis and lactate production within the gland1 epithelia (Figure S6g, S6i). This finding highlights the critical role of progesterone in enabling the epithelium to achieve an optimal metabolic state necessary for successful implantation.

### 2.5 Insufficient Lactate Causes Implantation Failure Through Impaired Immune Balance in Mice

**FIGURE 5:**
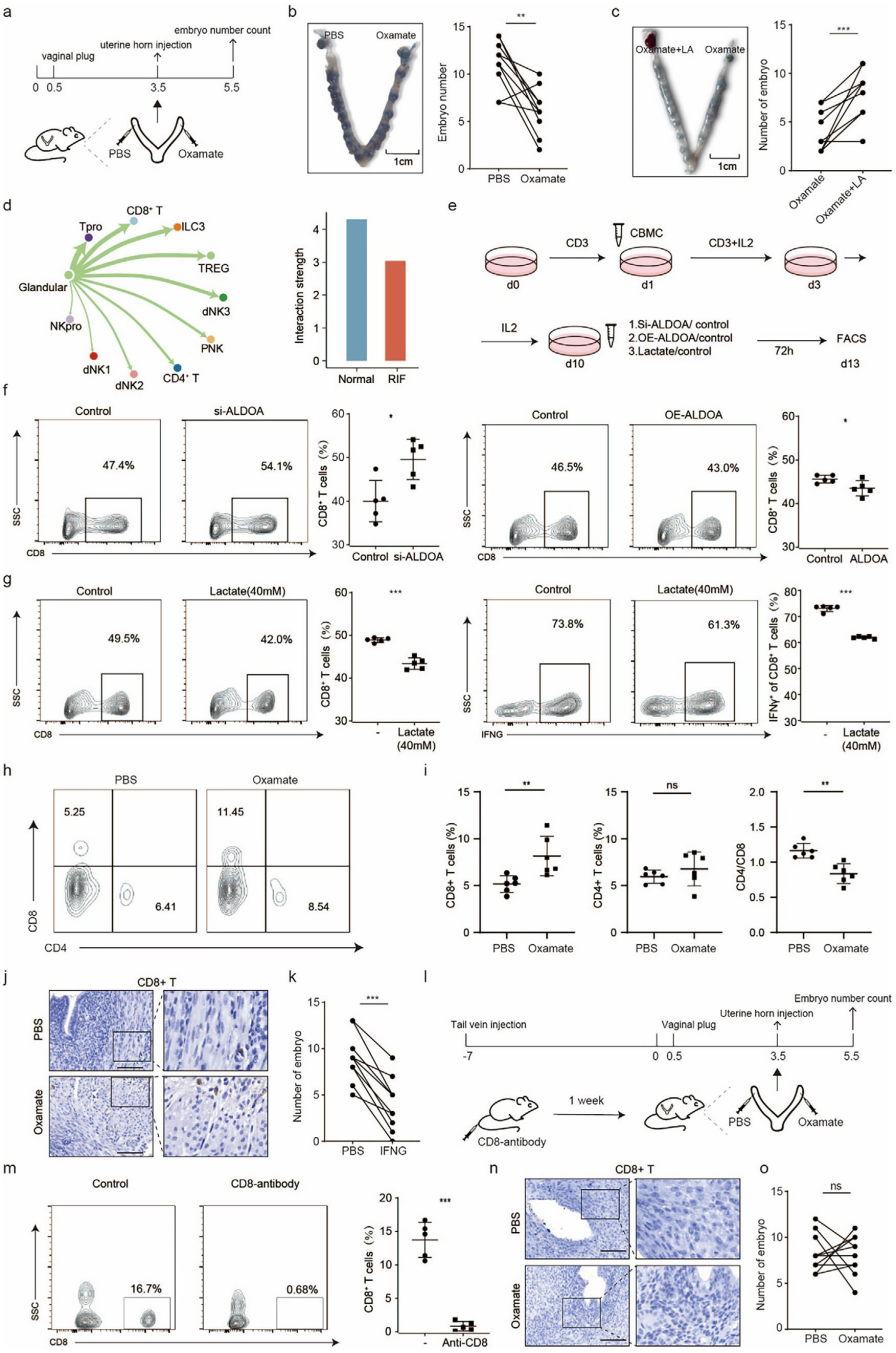
Insufficient lactate causes implantation failure through impaired immune balance. (a) Schematic of the in vivo experiment. (b) Left: Representative uteri treated with oxamate (left) or PBS (right) on Day 5.5. Right: Number of embryo implantation sites. *p* values were calculated by wilcoxon matched pairs signed rank test (n = 10 per group). **, *p* < 0.01. (c) Left: Representative uteri treated with oxamate + LA (left) and oxamate (right) on Day 5.5. Right: Number of embryo implantation sites. *p* values were calculated by wilcoxon matched pairs signed rank test (n = 10 per group). ***, *p* < 0.001. (d) Left: Circle plot showing the interaction weight networks from the glandular epithelium to lymphocytes. A thicker line represents a greater number of interactions and a stronger interaction weight/strength between the two cell types. Right: The interaction strength between the glandular epithelium and lymphocytes in the controls and patients with unexplained RIF. (e) Schematic of the in vitro experiment. (f) Flow cytometric analysis of CD8^+^ T‐cells among CD3^+^ T‐cells after incubation in the supernatants of control (−) and si‐ALDOA/overexpressing ALDOA ECC‐1 epithelia for 48 h. The data are presented as the mean ± s.d. and were analyzed by two‐tailed Student's *t*‐test. *, *p* < 0.05. (g) Flow cytometric analysis of CD8^+^ T‐cells among CD3^+^ T‐cells and IFNG^+^ cells among stimulated CD8^+^ T‐cells after incubation in the absence (−) or presence of lactate ECC‐1 epithelium for 48 h. The data are presented as the mean ± s.d. and were analyzed by two‐tailed Student's *t*‐test. ***, *p* < 0.001. (h) Flow cytometric analysis of CD8+ T‐cells and CD4+ T‐cells percentage in the mice uteri treated with oxamate or PBS. (i) The proportion of CD8+T‐cells, CD4+T‐cells, and the ratio of CD4/CD8. The data are presented as the mean ± s.d. and were analyzed by two‐tailed Student's t‐test (n = 6 per group). **, *p* < 0.01. (j) Immunohistochemical staining for the expression of CD8A in the endometria of mice injected with PBS or oxamate. Scale bar, 100 µm. (k) Number of embryo implantation sites in PBS and IFNG on Day 5.5. *p* values were calculated by wilcoxon matched pairs signed rank test (n = 10 per group). ***, *p* < 0.001. (l) Schematic of the in vivo experiment. (m) Flow cytometric analysis of CD8^+^ T‐cells in the peripheral blood on Day 5.5 of pregnancy. Left: control mice; right: mice subjected to anti‐CD8 treatment. The data are presented as the mean ± s.d. and were analyzed by two‐tailed Student's *t*‐test. ***, *p* < 0.001. (n) Immunohistochemical staining for the expression of CD8A in the endometria of mice treated with anti‐CD8 antibodies and injected with PBS or oxamate. Scale bar, 100 µm. (o) Number of embryo implantation sites in uteri injected with PBS or oxamate after anti‐CD8 therapy. *p* values were calculated by wilcoxon matched pairs signed rank test (n = 10 per group).

Given the reduced lactate production in patients with unexplained RIF, we investigated whether lactate in the uterine microenvironment is beneficial for implantation. To assess the impact of lactate on implantation in vivo, we performed experiments in ICR mice by injecting one side of the uterus with the lactate dehydrogenase inhibitor oxamate and the other side with phosphate‐buffered saline as a control (Figure 5a). The number of implantation sites was significantly lower on the oxamate‐injected side than on the control side (Figure 5b). Notably, the implantation defects caused by oxamate were reversed by the addition of exogenous lactate (Figure 5c). These findings suggest that sufficient lactate production is essential for embryo implantation.

Since lactate serves as a critical molecule in immune regulation [19], we hypothesized that the effect of lactate on embryo implantation may be achieved by affecting immune balance. To explore the relationship between lactate production and immune balance in RIF, we first evaluated the crosstalk between the gland1 epithelia and lymphocytes in our scRNA‐seq data. Strikingly, signalling from the gland1 epithelia to proliferative T cells and CD8^+^ T‐cells was the most common type of crosstalk (Figure 5d). The strength of the interaction between the gland1 epithelia and lymphocytes was weaker in the patients with unexplained RIF than in the controls (Figure 5d; Figure S7a,b). The interaction pathways were classified into 4 clusters based on similarities in their cellular communication network (Figure S7c). Notably, Cluster 1 is dominated by immune regulation pathways (e.g., SPP1, MIF, MK, and COMPLEMENT) and largely represents signalling from the gland1 epithelia to lymphocytes (Figure S7d,e). The pathways in Cluster 1 have been reported to be regulated by hypoxia and associated with glycolysis [20]. For example, MIF increases the synthesis of fructose 2,6‐bisphosphate and further increases cellular lactate production [21]. We inferred that lactate production in the endometrium might inhibit CD8^+^ T‐cells activation. This process promotes the establishment of immune balance in preparation for embryo implantation. However, the reduced lactate production in patients with unexplained RIF failed to produce this immune balance.

To further explore whether lactate can affect the proliferation and activation of CD8^+^ T‐cells, we treated naive T cells with the supernatant from si‐ALDOA ECC‐1 cells and measured the proportions of CD8^+^ T‐cells and IFNG^+^CD8^+^ T‐cells after 72 h (Figure 5e). The results revealed that higher proportions of CD8^+^ T‐cells and IFNG^+^CD8^+^ T‐cells were present in the si‐ALDOA group than in the control group (Figure 5f; Figure S8a). In contrast, there were lower proportions of CD8^+^ T‐cells under treatment with the supernatant from *ALDOA*‐overexpressing ECC‐1 cells (Figure 5f; Figure S8a). We also found that lactic acid inhibited the proliferation and function of CD8^+^ T‐cells, whereas hydrochloric acid did not, even at the same pH (Figure S8b). In addition, the proportions of CD8^+^ T‐cells and IFNG^+^CD8^+^ T‐cells were significantly decreased when naive T cells were treated with exogenous sodium lactate, suggesting that lactate is an important mediator of the effects of epithelial cells on the differentiation of CD8^+^ T‐cells and IFNG secretion (Figure 5g). Using flow cytometry and immunohistochemistry, we examined CD8^+^ T‐cells from the endometria of the intrauterine‐injected mice as mentioned above, and more CD8^+^ T‐cells were found in the oxamate‐injected group than in the control group (Figure 5h–j). To further explore the effect of IFNG on embryo implantation, we performed intrauterine injection with recombinant IFNG to verify its effect on embryo implantation. Compared with that on the control side, the number of implanted embryos on the side with IFNG injection was significantly lower (Figure 5k). Subsequent analysis of published embryo datasets revealed that the interferon‐gamma receptor (*IFNGR*) gene is expressed in embryonic cells across various developmental stages (Figure S8c) [22]. In addition, the expression of *IFNG* in endometrial CD8^+^ T‐cells decreased progressively from the proliferative phase to the secretory phase and reached its lowest level during the mid‐secretory phase (Figure S8d). Collectively, these results indicate that insufficient lactate production by the gland1 epithelia might impair the immune balance of CD8^+^ T‐cells and result in implantation failure. Additionally, CD8^+^ T‐cells may exert this effect on the embryo through the secretion of IFNG.

We next verified whether CD8^+^ T‐cells mediate the effect of LA on embryo implantation. Mice were treated with anti‐CD8 antibodies 1 week before mating, and mice treated with anti‐NK antibodies were used as positive controls. Oxamate and PBS were injected separately into the two sides of the uterus on Day 3.5 of pregnancy (Figure 5l; Figure S8e). The CD8^+^ T‐cells were almost entirely eliminated in the peripheral blood and endometrium of mice treated with anti‐CD8 antibodies on Day 5.5 of pregnancy (Figure 5m,n). Interestingly, no differences in embryo implantation numbers were observed between the two sides of the uterus after anti‐CD8 treatment (Figure 5o). However, the number of implantation sites was still lower on the side of the uterus injected with oxamate than on the control side after anti‐NK treatment (Figure S8f,g). These results suggest that CD8^+^ T‐cells mediate the effects of lactate on embryo implantation.

**FIGURE 6:**
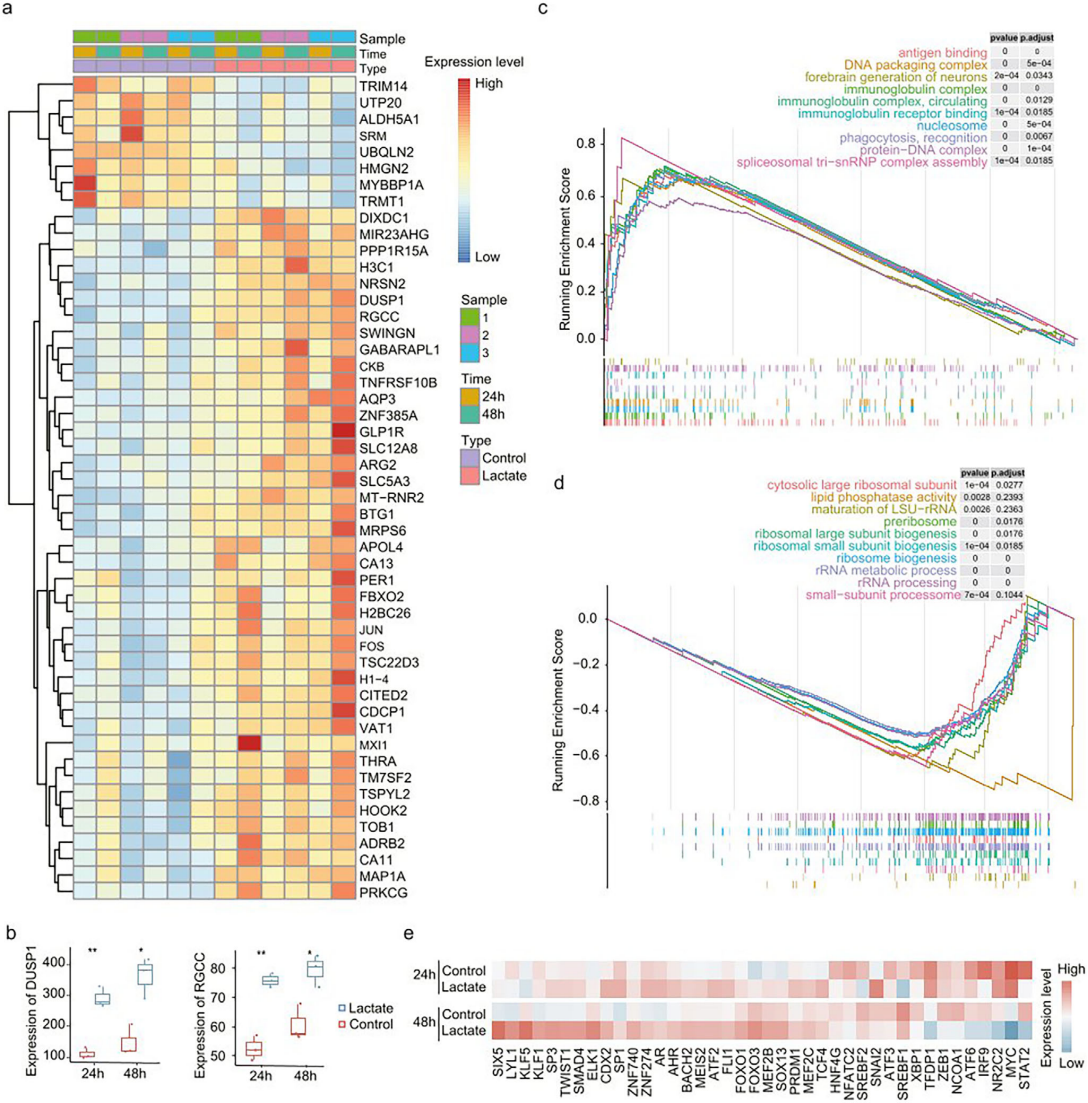
Effects of the glandular epithelium on lymphocytes. (a) Differential gene expression between sodium lactate‐treated and control T cells at 24 and 48 h. (b) Box plots showing the differences in the expression of DUSP1 and RGCC between the lactate treatment group and the control group at 24 and 48 h. Data are presented as median ± IQR. *P* values were calculated by two‐tailed Student's *t*‐test. *, *p* < 0.05; **, *p* < 0.01. (c,d) GSEA showing the upregulated (upper) and downregulated (down) differential pathways enriched in sodium lactate‐treated T cells. (e) Differential TFs between the lactate‐treated and control groups at 24 and 48 h.

To clarify how lactate regulates the proliferation and activation of CD8^+^ T‐cells, we cultured T cells in the presence or absence of exogenous sodium lactate. Considering the changes in the proportions of CD8^+^ T‐cells and IFNG^+^CD8^+^ T‐cells at 72 h, we collected the T cells at 24 and 48 h to obtain bulk RNA sequences. Differential gene expression analyses were conducted between the two groups at 24 and 48h. The results revealed that CD8^+^ T‐cells in the sodium‐lactate‐treated group expressed relatively high levels of genes such as *DUSP1*, *RGCC*, *JUN*, and *FOS* at both 24 and 48 h (Figure 6a). The upregulation of genes *FOS* and *JUN*, which regulate glycolysis, indicated altered metabolism in sodium‐lactate‐treated CD8^+^ T‐cells. *DUSP1* and *RGCC* are related to apoptosis and cell cycle regulation, respectively, and their upregulation explains the decreased proliferation of CD8^+^ T‐cells under sodium lactate treatment (Figure 6b). In addition, DEGs in the sodium‐lactate‐treated group were more enriched in the ‘antigen binding’ and ‘DNA packaging complex’ pathways but were less enriched in the ‘preribosome’ and ‘ribosome biogenesis’ pathways (Figure 6c,d). We subsequently used DoRothEA to infer transcription factor (TF) activities in T cells and compared differences between the patients with unexplained RIF and the controls. Intriguingly, we found that the lactate‐treated group presented lower expression of TFs that regulate *IFNG*, such as *STAT2* and IRF9 (Figure 6e) [23]. In conclusion, our study suggests that lactate can inhibit the proliferation of CD8^+^ T‐cells and the secretion of IFNG, with established immune balance further benefiting embryo implantation.

## 3 Discussion

Unexplained RIF is a substantial challenge and causes profound physical and mental harm to patients. Investigating its underlying mechanisms is crucial for developing new strategies for diagnosis and treatment. In this study, we explored the mechanism of unexplained RIF using the largest available scRNA‐seq dataset of the endometrium during the WOI, supplemented by external data and data from in vivo and in vitro experiments. We generated a cellular atlas of the human endometrium during the WOI. Strikingly, the endometria of the patients with unexplained RIF had greater proportions of CD8^+^ T‐cells with higher expression of IFNG than did those of the controls. Furthermore, we found that a subset glandular epithelia of the patients with unexplained RIF exhibited abnormal glycolysis and lower lactate production, which impaired the immune balance of CD8^+^ T‐cells and caused implantation failure.

A receptive endometrial environment requires adequate immune tolerance to protect the implanting embryo from maternal immune rejection. Maternal–fetal immune tolerance is a prerequisite for implantation and allows for the coexistence of embryonic and maternal cells. During the WOI, the maternal endometrium shifts to an immune‐tolerant state to protect the implanting embryo, a semi‐allograft, from maternal immune rejection [11]. Decidual NK cells are essential for the maintenance of immune tolerance and contribute to the remodeling of the spiral artery, which provides nutrients and oxygen to the fetus throughout pregnancy. Three main subsets of human decidual NK cells were identified by single‐cell RNA‐seq: NK1, NK2, and NK3. NK1 cells are thought to have fetal growth‐supporting activity, with high expression of glycolytic enzymes and cytoplasmic granule proteins [14]. NK2 and NK3 cells, which express high levels of *XCL1* and *CCL5*, potentially regulate placental extravillous trophoblast (EVT) invasion [24]. Previous studies have shown that NK1 expression is significantly reduced while NK3 expression is increased in the decidual immune cells of women with recurrent pregnancy loss [14]. A recent scRNA‐seq study on patients with RIF also suggested that the proportion of NK cells is decreased, possibly resulting from a decrease in a subset of endometrial epithelial cells with high levels of progesterone receptors, autophagy factors, and exosomes [13].

Our results revealed significant differences in the endometrial immune microenvironment during the WOI between the patients with unexplained RIF and the controls. While recognizing that inter‐individual variation in endometrial cellular composition is substantial, our analysis systematically compared the RIF and control groups and identified lymphocytes as showing the greatest relative increase in the RIF/control ratio, with this increase predominantly driven by CD8+ T‐cells. Among the T cell subsets, CD8^+^ T‐cells are characterized by high expression of cytotoxic granule genes such as NKG7 and GZMA, which may contribute to dysfunctional receptivity to embryo implantation [25]. The decreased proportions of lymphocytes and CD8^+^ T‐cells during the WOI might improve the establishment of immune balance and be conducive to embryo implantation, whereas the increased proportions of lymphocytes and CD8^+^ T‐cells in patients with unexplained RIF suggest a failure of immune balance establishment and poor endometrial receptivity. A recent study reported that B7‐H4 promotes fetal immune tolerance accompanied by CD8^+^ T cell exhaustion [26]. Additionally, the proliferation and activation of CD8^+^ T‐cells can be negatively regulated by T regulatory cells (Tregs), which are critical mediators of immune regulation and have been implicated in unexplained infertility [27, 28]. However, the precise mechanisms by which CD8+ T‐cells contribute to implantation failure remain unclear. It has been reported that excessive cytotoxic CD8^+^ T‐cells can induce fetal loss in mice under pro‐inflammatory conditions caused by infection or impaired balance [29]. Moreover, CD8^+^ T‐cells may also contribute to implantation failure through other indirect mechanisms, such as by disrupting decidualization and vascular remodeling [30]. Notably, CD8^+^ T‐cells in the patients with unexplained RIF expressed higher levels of IFNG, a classical cytotoxic protein of lymphocytes, with excessive levels being implicated in implantation failure [31]. Increased expression of IFNG in the endometria of women with recurrent miscarriage has also been reported [32]. Our findings reveal an immune imbalance in patients with unexplained RIF and highlight the importance of endometrial CD8^+^ T‐cells in implantation.

We also observed abnormal glycolysis and insufficient lactate production in the endometrial glandular epithelia of patients with unexplained RIF. A key characteristic of the human endometrium when entering the WOI is the abrupt and discontinuous transcriptomic activation of the epithelium [16]. In our study, among the seven endometrial cell types, the glandular epithelium had the greatest number of DEGs shared between the patients with unexplained RIF and the controls. The normal development of glands is essential for pregnancy establishment. The uterine glands synchronize embryo‐endometrial interactions and coordinate embryo implantation, thereby ensuring embryo viability and pregnancy success [33]. Our results further suggest that among all cell types in the endometrium, the metabolism of the glandular epithelium is the most active. Metabolic regulation plays an important role in endometrial receptivity and embryo implantation [34]. For example, arginine biosynthesis, which was differentially enriched in patients with unexplained RIF, has been reported to be beneficial for embryo implantation [35]. Many energy metabolic pathways were high enriched in the glandular epithelium. Glucose, lipids, and proteins are the three major energy‐providing substances, among which glucose is the primary source of energy for most cells, especially for cell growth and activities [36]. In general, glucose is taken up by cells via the glucose transporter type 1 (*GLUT1*) and metabolized into pyruvate, which further enters the mitochondrial tricarboxylic acid (TCA) cycle to provide energy through oxidative phosphorylation. However, under conditions of hypoxia, hypoxia‐inducible factor 1 (*HIF‐1*) down‐regulated the activity of pyruvate dehydrogenase indirectly and restrict the oxidation of pyruvate, which converts to be used to product lactate instead. Compared with those of the controls, the glandular epithelia in the patients with unexplained RIF exhibited abnormal glycolysis and reduced lactate production, with lower LDHA and SLC16A3 expression. Abnormalities in glycolysis have also been reported in other endometrial diseases, such as endometrial cancer [37]. While these multi‐level validations collectively demonstrate dysregulated lactate metabolism in RIF endometria, we recognize that large‐scale targeted metabolomic studies in well‐characterized unexplained RIF patient cohorts remain an important future direction to directly quantify the magnitude and heterogeneity of lactate alterations across diverse RIF populations [38].

Notably, the progesterone‐mediated attenuation of estrogen‐driven proliferation in the endometrial epithelium is a prerequisite for successful implantation [39]. Progesterone promotes the transition of the endometrial epithelium into a mature and receptive state, rendering it suitable for embryo implantation [40]. During the WOI, progesterone levels increase dramatically, activating the MYC and PI3K‐AKT signalling pathways. These pathways subsequently induce the activation of hypoxia‐inducible factor 1 (HIF1A) and create a hypoxic environment, which regulates the expression of enzymes involved in glycolysis. Under hypoxic conditions, glucose metabolism switches from OXPHOS to lactate production, and pyruvate is converted into lactate by LDHA. However, our results indicated that the unexplained RIF group exhibited no significant hormonal or metabolic abnormalities at baseline. Unexplained implantation failure is unlikely to be attributed to a single factor. Rather, any factors contributing to abnormal epithelial cell development could represent potential causes.

Notably, we found that the expression of *ALDOA* in glandular epithelial cells was also lower in patients with unexplained RIF and that lower expression further reduced the production of lactate. The enzyme ALDOA accelerates glycolysis by catalyzing the conversion of fructose‐1,6‐bisphosphate to glyceraldehyde‐3‐phosphate (G3P) and dihydroxyacetone phosphate [41]. Previous studies have suggested that many metabolic pathways could be regulated by ALDOA. For example, ALDOA is a target of HIF1A and induces fatty acid and nucleotide biosynthesis during tumorigenesis by accelerating glycolysis [42]. Furthermore, lactate was identified as the major target in ALDOA‐mediating metabolism under hypoxia. Local lactate shuttles are activated in the decidua and play important roles in supporting early pregnancy [15]. Furthermore, lactylation is a newly identified post‐translational modification reported in studies of the endometrium. Lactylation was originally discovered in human histones [43]. Lactate can remodel uterine receptivity by inducing endometrial H3K18 lactylation and regulating redox homeostasis and the apoptotic balance to ensure successful implantation [44]. Interestingly, lactylation is common to glycolytic enzymes and conserved in ALDOA. The widespread occurrence and appreciable abundance of the lactylation of ALDOA suggest its important role in maintaining the lactate microenvironment [45]. Lactate levels regulate ALDOA by lactylation, resulting in the formation of a feedback loop for lactate production.

The mammalian blastocyst also exhibits a high capacity for aerobic glycolysis and releases significant amounts of lactate into the endometrial micro‐environment. The lumen of the uterus is a hypoxia environment, creating a condition for lactate production. In addition, the implantation site of embryo is relatively anoxic cause the absence of maternal vasculature during the early phases of implantation [46]. However, the blastocyst converts much of the glucose consumed to lactate, even in the presence of oxygen. This ability of the blastocyst may allow it to adapt to the high oxygen environment brought about by the subsequently established maternal vasculature, allowing it to continue to produce lactic acid. The high lactate created by the blastocyst is thought to contribute to facilitate trophoblast invasion, remodel vasculature, and modulate the local immune, thereby facilitating successful implantation. In addition, lactic acid can also be produced by lactobacilli, which are dominant in the healthy female reproductive tract. Uterine epithelial acidification as an important phenomenon during embryo implantation [47]. However, the pH of the endometrial fluid is not correlated with the endometrial microbiota, suggesting other biochemical effects in the endometrium, where the embryo will adhere and develop [48]. Furthermore, our results revealed that lactic acid inhibited the proliferation and function of CD8^+^ T‐cells, whereas hydrochloric acid did not, even at the same pH, indicating the additional role of lactate in immune regulation and embryo implantation. These findings highlight the important role of lactate in embryo implantation.

We suggest that lactate in the endometrium blunts immune rejection to promote embryo implantation. Spatially, immunofluorescence analysis revealed that CD8+ T‐cells are distributed in three distinct patterns within the endometrium: (1) directly adjacent to glandular epithelium, enabling potential local cell‐cell interactions and direct lactate sensing; (2) dispersed throughout the stromal compartment, where they may encounter lactate secreted into the interstitial fluid; and (3) localized near vasculature, potentially intercepting lactate delivered systemically. An increasing number of studies have revealed the role of lactate in regulating immune functions [49]. Immunosuppressive networks have been established in the presence of lactate to promote immune tolerance [50]. Previous studies have indicated that lactate acts as a mediator between metabolic reprogramming and immunosuppression [51]. Lactate can induce the aggregation of Tregs and initiate their immunosuppressive effects [52]. Lactate levels are also associated with T cell and NK cell activity [49]. However, a limitation of our study is that the number of Tregs was not sufficient for further investigation via scRNA‐seq, although we observed a direct effect of lactate on CD8^+^ T‐cells in vitro. We link altered endometrial glucose metabolism and immune balance and show that increased lactic acid production in the glandular epithelium impairs the proliferation of CD8^+^ T‐cells and the production of cytokines, particularly IFNG, thereby inhibiting embryo immunosurveillance and promoting implantation. However, the insufficient lactate production in patients with unexplained RIF interrupts these processes. Furthermore, despite collecting comprehensive clinical and lifestyle data, we could not definitively pinpoint the specific upstream factors driving the observed alterations in glandular lactate metabolism. This limitation may constrain the interpretation of lactate dysregulation in RIF. Finally, although our sample size was adequate for initial discovery and validation, it might not encompass the full heterogeneity of RIF. Therefore, larger, multi‐center cohort studies are warranted to validate and extend these findings. We believe these acknowledgments offer a more balanced perspective and better define the scope and generalizability of our conclusions.

Our study elaborates on the potential pathogenesis of unexplained RIF by investigating transcription in the human endometrium. We propose that insufficient endometrial lactate might impair embryo immune balance by CD8^+^ T‐cells and further result in implantation failure. These findings provide new insight into the diagnosis and treatment of unexplained RIF.

## 4 Methods

### 4.1 Sample Characteristics

This study was conducted at four hospitals in China: the First Affiliated Hospital of Nanjing Medical University, the Suzhou Hospital Affiliated with Nanjing Medical University, the Shengjing Hospital of China Medical University, and the Third Affiliated Hospital of Zhengzhou University. Considering the recent requirements for embryo transfer and the patients at our hospitals, we recruited patients with recurrent implantation failure, defined as women under 40 years of age who failed to achieve pregnancy after three or more IVF/ICSI attempts or who failed after two attempts and whose cumulative number of transferred embryos was no less than four with the use of cleavage‐stage embryos and no less than two with the use of blastocysts. The candidates for the control group were women requesting their first IVF/ICSI due to male factor infertility. All participants provided written informed consent. To investigate the mechanism of unexplained RIF, we excluded patients with RIF with identified conditions such as endometritis, uterine malformation and adenomyosis. We also excluded participants with specific conditions from the control group. Participants with disease associated with RIF were excluded according to medical records and laboratory tests, including chromosome examination (chromosomal abnormalities), reproductive hormone and thyroid function assessments (endocrine diseases), oral glucose tolerance and biochemical tests (metabolic diseases), cancer antigen 125 and carbohydrate antigen 19‐9 tests (for endometriosis), evaluations of homocysteine, D‐dimer, and adiponectin levels (to evaluate the prethrombotic state), and tests for immune markers such as anti‐β2‐glycoprotein 1, anticardiolipin, and antinuclear antibodies (for immune diseases). Questionnaires and clinical information were collected from couples at baseline and at follow‐up. In addition, biological samples such as peripheral blood samples from both partners, endometrial samples, vaginal swabs, and cervical swabs were collected at scheduled times. In the subsequent process, the candidates in control group were required to confirm a clinical pregnancy otherwise they would be excluded from control group. Clinical pregnancy is defined as observation of an intrauterine gestational sac by a transvaginal ultrasound scan around 35 days after embryo transfer (Figure 1a).

During the collection of samples representing the phases of WOI, the women did not use glucocorticoids, antibiotics, or vaginal medications; had not engaged in sexual intercourse or vaginal douching within 5 days; had not received cervical treatment within 1 week; and had used contraception for the past month. Endometrial biopsy was conducted 5 days after ovulation (LH + 7, during the WOI). In detail, Plan A was used for patients with a regular menstrual cycle and normal ovulation: (1) The day after peak LH in blood or urine was identified as Day 0 (D0) in natural cycle monitoring. Patients were asked to take Duphaston 10 mg BID orally for 5 days from D1, and samples were collected in the morning on D6. (2) When the follicle diameter reached 18–20 mm under B‐mode ultrasound monitoring and HCG levels were in the range of 5000—10 000 IU/L, the second day was identified as D1, when ovulation was confirmed under B‐ultrasound monitoring. Subsequent treatment was the same as above until sample collection. Plan B was used for patients with an irregular menstrual cycles and abnormal ovulation: From the third to fifth days of menstruation, 2.5 mg of letrozole QD taken orally was administered for a total of 3–5 days, and HMG was added as appropriate until the follicle diameter reached 18–20 mm and HCG levels were in the range of 5000—10 000 IU/L. The second day was identified as D1, when ovulation was confirmed under B‐ultrasound monitoring. Subsequent treatment was the same as above until sample collection. If the patients under Plan A did not meet the sampling standards, they were transferred to Plan B in the second month.

To elucidate the mechanism underlying endometrial abnormality in patients with unexplained RIF, endometrial biopsies from 11 patients with unexplained RIF and 10 controls were used for single‐cell RNA sequencing (scRNA‐seq) (Figure 1a). The endometrial samples used for scRNA‐seq were obtained from patients at the First Affiliated Hospital of Nanjing Medical University or the Suzhou Hospital Affiliated with Nanjing Medical University. Moreover, endometrial samples from 48 patients with unexplained RIF and 19 controls were used to perform bulk RNA sequencing and passed quality control.

### 4.2 Statistical Analysis

Data are presented as the mean ± standard deviation (s.d.) or the median and interquartile range (IQR). The choice of statistical test was based on the number of groups and the presence of a normal distribution. Unless otherwise specified, a two‐tailed Student's *t*‐test and nonparametric Mann‐Whitney U‐test (Wilcoxon rank‐sum test, two‐sided) were used to compare means or medians between groups as indicated. Significance is indicated as *p < 0.05, **p < 0.01, and ***p < 0.001. Throughout the text, the number of independent biological samples or animals/groups in the same experiment is indicated by *n* = x, and the number of independent experiments is indicated by N  =  x. No statistical methods were used to predetermine sample sizes; however, our sample sizes are similar to those reported in previous publications. Animals/samples were randomly assigned to the various experimental groups and single blinding was applied in animal models. No animals were excluded from the analyses. Statistical analysis was performed using GraphPad Prism v8.

### 4.3 Tissue Dissociation and Preparation

Fresh endometrium of the uterus was washed with Hanks’ Balanced Salt Solution (HBSS) three times and minced into 1–2 mm pieces. The pieces were subsequently digested with 2 mL of GEXSCOPE Tissue Dissociation Solution (Singleron) at 37°C for 15 min in a 15 mL centrifuge tube with sustained agitation. After digestion, 40‐micron sterile strainers were used to filter the samples, and the samples were centrifuged at 1000 rpm for 5 min. The supernatant was subsequently discarded, and the sediment was resuspended in 1 mL of PBS (HyClone). The mixture was then centrifuged at 500 × g for 5 min and suspended in PBS. The sample was stained with trypan blue (Sigma), and cell viability was evaluated microscopically.

### 4.4 Single‐Cell RNA Sequencing

Single‐cell suspensions were converted to barcoded single‐cell sequencing (scRNA‐seq) libraries by using the Chromium Single‐Cell Library, Gel Bead & Multiplex Kit (10× Genomics) following the manufacturer's instructions. Briefly, cells were partitioned into gel beads in emulsion in the Chromium Controller instrument, where cell lysis and barcoded reverse transcription of RNA occurred. Libraries were prepared using 10× Genomics Library Kits and sequenced on an Illumina NovaSeq 6000 with 150 bp paired‐end reads.

### 4.5 scRNA‐Seq Data Processing

Raw reads were processed to generate gene expression profiles using Cell Ranger v.3.0.2. Reads from the 10× library were mapped to GRCh38 with Ensembl version 92 gene annotation. Reads with the same cell barcode, UMI and gene were grouped together to calculate the number of UMIs per gene per cell. The UMI count tables of each cellular barcode were used for further analysis. We used the package Seurat (V4.1.3) for quality control and downstream analysis of our single‐cell RNA‐seq data. All functions were run with default parameters unless otherwise specified. Before incorporating a sample into our merged dataset, we individually inspected the cell‐by‐gene matrix of each as a Seurat object. We calculated the number of unique genes detected in each cell (nFeature_RNA), the total number of molecules detected within a cell (nCount_RNA), and the percentage of reads that mapped to the mitochondrial genome (percent mt). Low‐quality cells were filtered out from the analysis using the following parameters: nFeature_RNA 200–7000, nCount_RNA< 40 000, and percent mt < 30%.

We then used SCTransform to normalize the data, RunPCA to perform dimension reduction, FindNeighbors and FindClusters to cluster the cells, and RunUMAP to visualize the data. Cell cycle analysis was performed with CellCycleScoring. The cell cycle phase (G1, S, or G2/M) of each cell was estimated based on its G2/M and S phase marker expression. We used Harmony (v.0.1.0) to reduce the number of technical batch effects when integrating samples. The main cell types in the dataset were identified and labelled by manual examination of typical marker genes from the literature. We removed the potential doublet cells that expressed markers typical for two cell types. After quality control, a total of 179 857 cells comprising 86 075 cells from the controls (n = 10) and 93 782 cells from the patients with unexplained RIF (n = 11) were retained for downstream analysis. We subsequently performed clustering analysis and identified the cell types of every cluster via well‐defined cell‐type‐specific marker genes. Fibroblasts (Fib) were defined by high expression of stromal extracellular‑matrix genes *DCN*, *LUM* and *COL1A1*, whereas smooth muscle cells (SMC) showed specific expression of markers *RGS5* and *ACTA2*. Endothelial cells (End) were identified by selective expression of *CLDN5*, *PECAM1* and *VWF*. The lymphocyte cluster (Lym) expressed T/NK‑cell genes *CD3E*, *CCL5* and *KLRD1*, and was clearly separated from the macrophage/monocyte cluster (Mac), which instead expressed myeloid markers CD68, *CD14* and *MS4A6A*. Non‑ciliated epithelial cells (Epi) were annotated based on strong epithelial markers *EPCAM*, *KRT8* and *UCA1*, and the ciliated epithelial subset (CEpi) was further distinguished by the additional, highly restricted expression of cilia‑associated genes *PIFO*, *FOXJ1* and *DYDC2*. As a result, seven cell types were identified in the endometrium during the WOI. To explore the changes in the proportion of every cell type in the patients with unexplained RIF, we calculated the ratio of cells from these patients to cells from the controls in the seven cell types. Considering that lymphocytes play a vital role in implantation and that their proportion sharply increases in patients with unexplained RIF, we performed further clustering analysis and more detailed annotation within lymphocytes according to known markers and the cluster‐specific genes identified by FindAllMarkers. To investigate the changes in the function of each cell type in patients with unexplained RIF, FindMarkers was applied to identify genes that were differentially expressed between the patients with unexplained RIF and the controls. As the epithelium presented the greatest number of DEGs and is closely related to the receptivity of the endometrium, we performed further clustering analysis and more detailed annotation within the epithelial cells. The reliability of the annotations was further confirmed by identifying highly variable genes within each annotated cell population, as well as by considering the clustering results and the spatial distribution of each cluster on UMAP projections, which together guided the precise annotation of cellular subsets. Finally, three epithelial subsets were identified and used for further analysis.

### 4.6 Gene Set Variation Analysis (GSVA)

Gene set variation analysis (GSVA) can estimate the relative enrichment of pathways across cells and is used to observe the variation in the activity of a set of genes corresponding to a particular biological condition [53]. Pathway analyses were predominantly performed on the 50 hallmark pathways described in the molecular signature database. To reduce pathway overlaps and pathway redundancies, each gene set associated with a pathway was trimmed to contain only unique genes, and all genes associated with two or more pathways were removed. Next, to assign pathway activity estimates to individual cells, we applied GSVA using standard settings, as implemented in the GSVA package (version 1.42.0).

### 4.7 Cell Differentiation Trajectory Inference

Monocle 3 was used to infer the cell differentiation trajectories [54]. The package organizes cells into potentially discontinuous trajectories according to their gene expression. Then, optional statistical tests were used to identify differentially expressed genes across the constructed trajectories, which could be grouped into different coregulated modules that can reveal fate‐specific genes.

### 4.8 Cell‐Cell Interaction Analysis

The cell‐cell interactions between the glandular epithelium and lymphocytes were evaluated using CellChat (version 1.5.0) [55]. CellChat takes gene expression data as user input to model the probability of cell‐cell interactions by integrating gene expression data with the existing ligand‐receptor interaction database. The analysis follows the default pipeline. Normalized count data from each condition were used to create a CellChat object, and the recommended preprocessing functions for the analysis of individual datasets with default parameters were applied. To compare the differences in intercellular communication between the endometrial samples from the controls and the patients with unexplained RIF, we further analyzed them together via joint manifold learning and classification of the inferred communication networks based on their functional similarity.

### 4.9 Single‐Cell Metabolic Analysis

To analyze metabolism via scRNA‐seq, scMetabolism (version 0.2.1) was used to quantify the metabolic activity in single cells. scMetabolism was prepopulated with 85 KEGG pathways and 82 Reactome entries [56]. The R package utilized the vision algorithm to score each cell in every metabolic pathway based on the conventional single‐cell matrix file.

### 4.10 Real‐Time PCR Analysis

Total RNA from human endometrial tissues was extracted using TRIzol reagent (Invitrogen) and reverse transcribed into cDNA using the HiScript III RT SuperMix for RT‐qPCR (+ gDNA wiper) (R323‐01, Vazyme Biotech). Real‐time PCR was performed using the ChamQ SYBR RT‐qPCR Master Mix (Low ROX Premixed) (Q331‐AA, Vazyme Biotech) on an iCycler RT‐PCR Detection System (Bio‐Rad Laboratories). The β‐actin gene was used as an internal control. Each assay was performed in triplicate for each sample. The primer sequences used for real‐time PCR are listed in Supplementary Table S7.

### 4.11 IHC

Newly collected endometrial tissues were fixed in 10% buffered formalin for paraffin embedding and sectioning. After deparaffinization and rehydration, endogenous peroxidase activity was blocked by incubation in 3% hydrogen peroxide in methanol for 20 min. Antigen retrieval pretreatment was carried out by boiling the sections in 0.01 M citrate buffer, pH 6.0, for 10 min. Immunohistochemical analyses were performed using SPlink Detection Kits (ZSGB‐Bio) with specific antibodies overnight at 4 °C. Negative controls were incubated with nonimmune IgGs (Table S8).

### 4.12 Flow Cytometry

T cells from human cord blood were used for analysis. For surface staining, the cells were incubated with the indicated antibodies on ice for 30 min and washed with staining buffer (2% FBS in PBS with 0.1% NaN_3_ and 1 mM EDTA). For intracellular staining, the cells were fixed and permeabilized via a BD Cytofix/Cytoperm Fixation/Permeabilization Kit after stimulation with Leukocyte Activation Cocktail (BD) for 4 h. Stained cell populations were acquired on a Beckman Cytoflex, and the data were analyzed with FlowJo v10 software.

### 4.13 Extracellular Acidification Rate Analysis

The extracellular acidification rate (ECAR), a measure of glycolysis, was measured in real time with a Glycolysis Stress Test Kit using the Seahorse XF96 Analyser (Agilent Technologies) following the manufacturer's instructions [57]. Briefly, the test consisted of four consecutive stages: basal (without drugs), glycolysis induction (10 mM glucose), maximal glycolysis induction (1 µM oligomycin), and glycolysis inhibition (50 mM 2DG). The rate of glycolysis is defined as the change in the ECAR between post‐glucose addition and baseline, and glycolytic capacity is defined as the change in the ECAR between post‐oligomycin addition and baseline.

### 4.14 Cell Culture

The human endometrial cancer cell line ECC‐1 endometrial epithelial cells (U80244, YOBIBIO) were used for in vitro experiments. The cells were maintained in RPMI‐1640 medium (Gibco, Carlsbad, CA, USA) containing 10% FBS (Gibco, USA), 1% penicillin/streptomycin (Gibco, USA), and DMEM (Gibco, USA) supplemented with 10% FBS and 1% penicillin/streptomycin. The cells were maintained at 37°C in a humidified incubator with 5% CO_2_.

### 4.15 Mice and In Vivo Experiments

ICR female mice (8–10 weeks) were naturally mated with proven fertile ICR stud males overnight, and the presence of a copulatory plug was designated 0.5 days post coitum (dpc). At 3.5 dpc, intrauterine injections were performed using a dorsal approach. Specifically, one uterine horn of the female mice was slowly injected with 10 µL of PBS using a 26‐gauge Hamilton syringe (No. 80300), while the other side received 10 µL of 25 mg/mL oxamate. Different markers were used on each side of the mouse fur to label the two treatments. At 5.5 dpc, the treated mice were sacrificed to count the number of implantation sites in each uterines horn, to assess the effect of the two treatments on implantation. Similar approaches were conducted in other experimental interventions. In the rescue experiment, one uterine horn of female mice were slowly injected with 10 µL of 25 mg/mL oxamate, while the other side received 10 µL of a mixture of 25 mg/mL oxamate and 1 mM lactic acid. In the IFNG treatment experiment, one side uterine horns of female mice was slowly injected with 10 µL PBS, while the other side was with 10 µL of 10 ng/mL IFNG. For antibody treatment experiments, 200 µg of anti‐CD8α (BioXCell, Cat# 53–6.7) or anti‐NK1.1 (BioXCell, Cat# PK136) was injected intravenously into the tail vein 1 week prior to mating to deplete the corresponding cells throughout the body. At 3.5 dpc, tail blood was collected to verify the depletion effect of antibodies via flow cytometry. Simultaneously, one side uterine horn of female mice was slowly injected with 10 µL of PBS, while the other side was with 10 µL of 25 mg/mL oxamate. At 5.5 dpc, the mice were sacrificed and counted the numbers of implantation sites in each uterine horn.

### 4.16 Ethical Approval

Ethical approval for the cohort study was obtained from the institutional review board of Nanjing Medical University (No. NJMUIRB (2016) 311). All animal experiments complied with the rules and guidelines of the Animal Care and Use Committee of Nanjing Medical University, and approval was obtained for all experimental protocols (No. IACUC‐2312047).

## Author Contributions

Z.H., Y.H., Y.G. and H.L. initiated, conceived, and supervised the study. Y.H. and K.K. performed the bioinformatics and statistical analyses and drafted the manuscript with Y.G. K.K., M.A. and X.W. conducted the experiments with J.C. and Z.H., Y.L., X.W., Y.G., and H.M. participated in the design and organization of the CNBC cohort study; F.D., Q.M., Y.G., J.T., B.H., C.T. and Q.Z. assisted in sample collection and clinical information organization. The manuscript was revised by all the authors.

## Funding

This project was supported by the National Key Research & Development (R&D) Program of China (Nos. 2024YFC2706900, 2025YFC2708001, 2021YFC2700600), the Science Fund for Creative Research Groups of the National Natural Science Foundation of China (No. 82221005).

## Conflicts of Interest

The authors declare that they have no conflicts of interest.

## Data and Materials Availability

The raw scRNA‐Seq data used in this study have been deposited in the NCBI GEO database under accession number GSE248784 at https://www.ncbi.nlm.nih.gov/geo/query/acc.cgi?acc=GSE248784 (enter token uvqboaqgxzgbnit). Additional single‐cell transcriptomes of endometrial biopsies during the menstrual cycle were downloaded from the Gene Expression Omnibus with GSE111976. Single‐cell transcriptomes of endometrial organoids response to ovarian hormones were downloaded from ArrayExpress with E‐MTAB‐10283.

## Supporting information




**Supporting File 1**: advs74583‐sup‐0001‐FigureS1‐S8.doc.

**Supporting File 2**: advs74583‐sup‐0002‐TableS1‐S8.xls.
