# Supplementary material for: Endometrial Epithelial Lactate Deficiency Drives CD8+ T‐Cells Dysregulation in Unexplained Recurrent Implantation Failure
Source: Adv Sci (Weinh). 2026 Feb 27;13(26):e24090. doi: 10.1002/advs.202524090 (PMC13159129; doi:10.1002/advs.202524090)
Supplement: Supplementary file 1 — Supporting File 1: advs74583‐sup‐0001‐FigureS1‐S8.doc. [file ADVS-13-e24090-s001.doc]

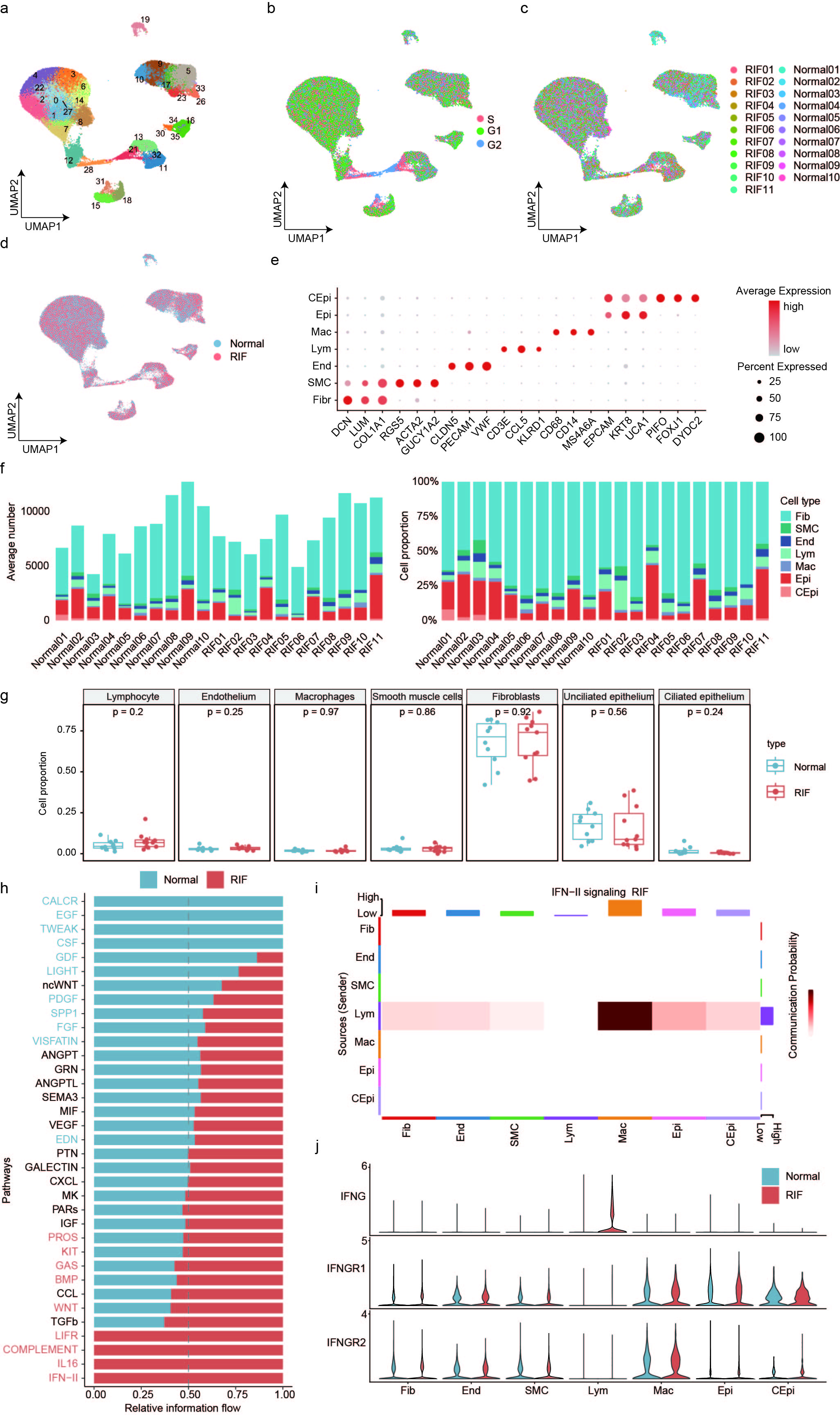


**Supplementary Fig. S1 | Cellular landscape of the endometrium during the WOI in patients with unexplained RIF. a** UMAP plot with 35 clusters (using Seurat shared nearest neighbour (SNN) for cluster identification). **b** UMAP representations coloured by the cell cycle. **c** UMAP representations coloured by participants (10 controls and 11 patients with unexplained RIF). **d** UMAP visualization of endometrial cells from the controls and patients with unexplained RIF. **e** Expression of classical marker genes across the seven major cell types. **f** Cell number and relative proportions of 7 cell clusters in each sample. **g** The proportions of seven major cell types from the controls and patients with unexplained RIF. Data are presented as median ± IQR. P values were calculated by Mann-Whitney U test (Normal, n = 10; RIF, n = 11). **h** All significant signalling pathways were ranked based on their differences in relative information flow within the inferred networks between the controls and patients with unexplained RIF. The top signalling pathways coloured blue were more enriched in the controls, the middle one’s coloured black were equally enriched in the controls and patients with unexplained RIF, and the bottom ones coloured red were more enriched in the patients with unexplained RIF. **i** Heatmap of IFNG signalling pathway components in RIF. The communication probability of the signalling pathway was computed by summarizing the probabilities of its associated ligand–receptor pairs. The darker the colour, the greater the communication probability was between the two cell types. **j** Violin plot showing the expression patterns of signalling genes involved in the inferred IFNG signalling network. The normalized expression levels of the controls and patients with unexplained RIF are shown in the violin plot.

**
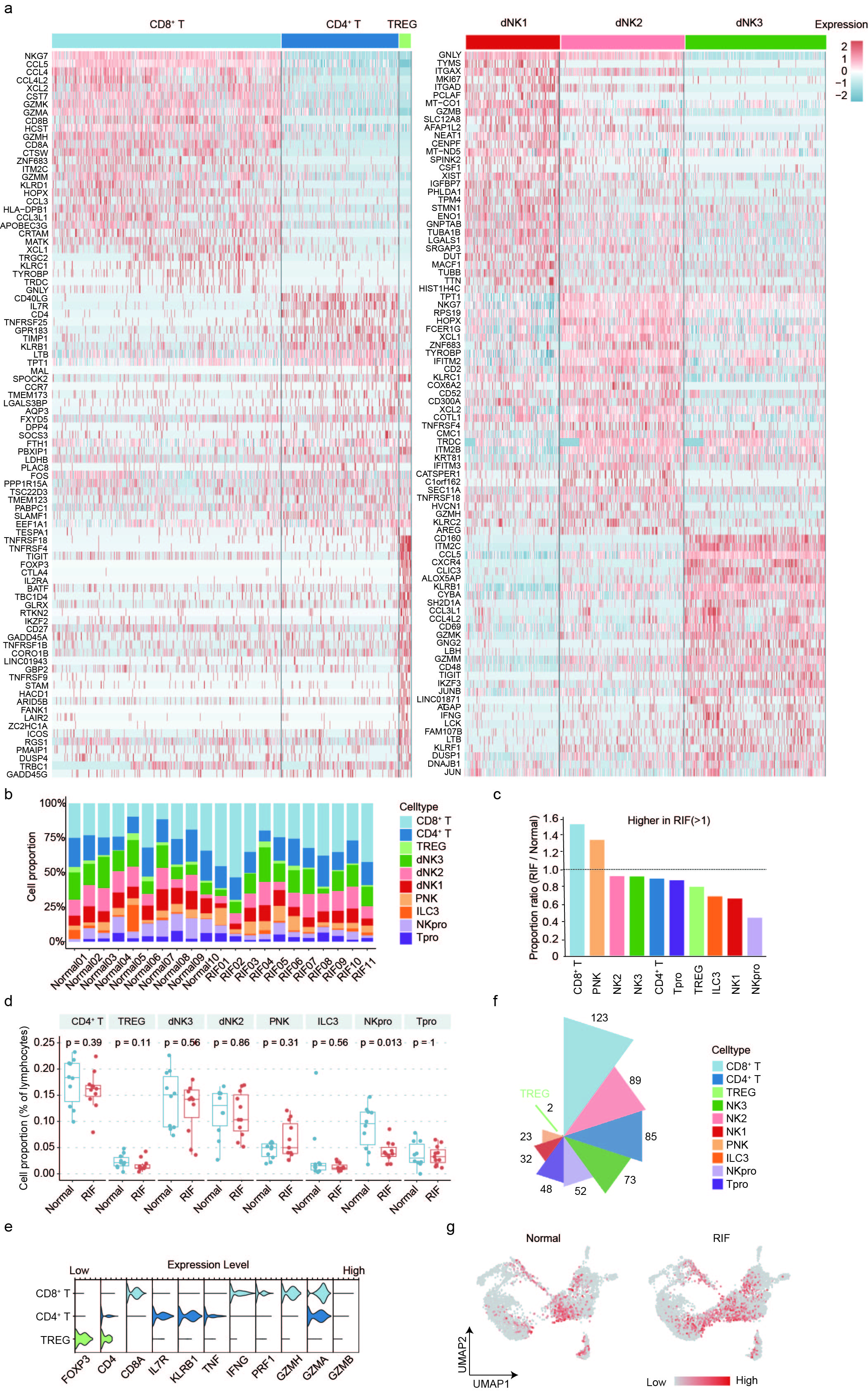
**

**Supplementary Fig. S2 | Increased proportions of lymphocytes and CD8^+^ T-cells in patients with unexplained RIF. a** Heatmap showing the expression signatures of the top 30 specifically expressed genes in each lymphocyte subset; the value for each gene is the row-scaled Z score. **b** Relative proportions of lymphocyte subsets in each sample. **c** Bar plot showing the differences in the proportions of lymphocyte subsets between the endometria of the controls and patients with unexplained RIF. **d** The proportions of CD4^+^ T cell, Tregs, dNK3 cell, dNK2 cell, PNK cell, ILC3s, NK-pro cell and T-pro cell subsets among the lymphocytes from the controls and patients with unexplained RIF. Data are presented as median ± IQR. *P* values were calculated by two-sided unpaired Mann–Whitney *U* test (Normal, n = 10; RIF, n = 11). **e** Violin plot showing the expression of cytotoxic and proinflammatory genes in the three subsets of endometrial T cells. **f** The number of differentially expressed genes in each lymphocyte subsets. **g** UMAP distribution of IFNG in the endometria of the controls and patients with unexplained RIF.

**
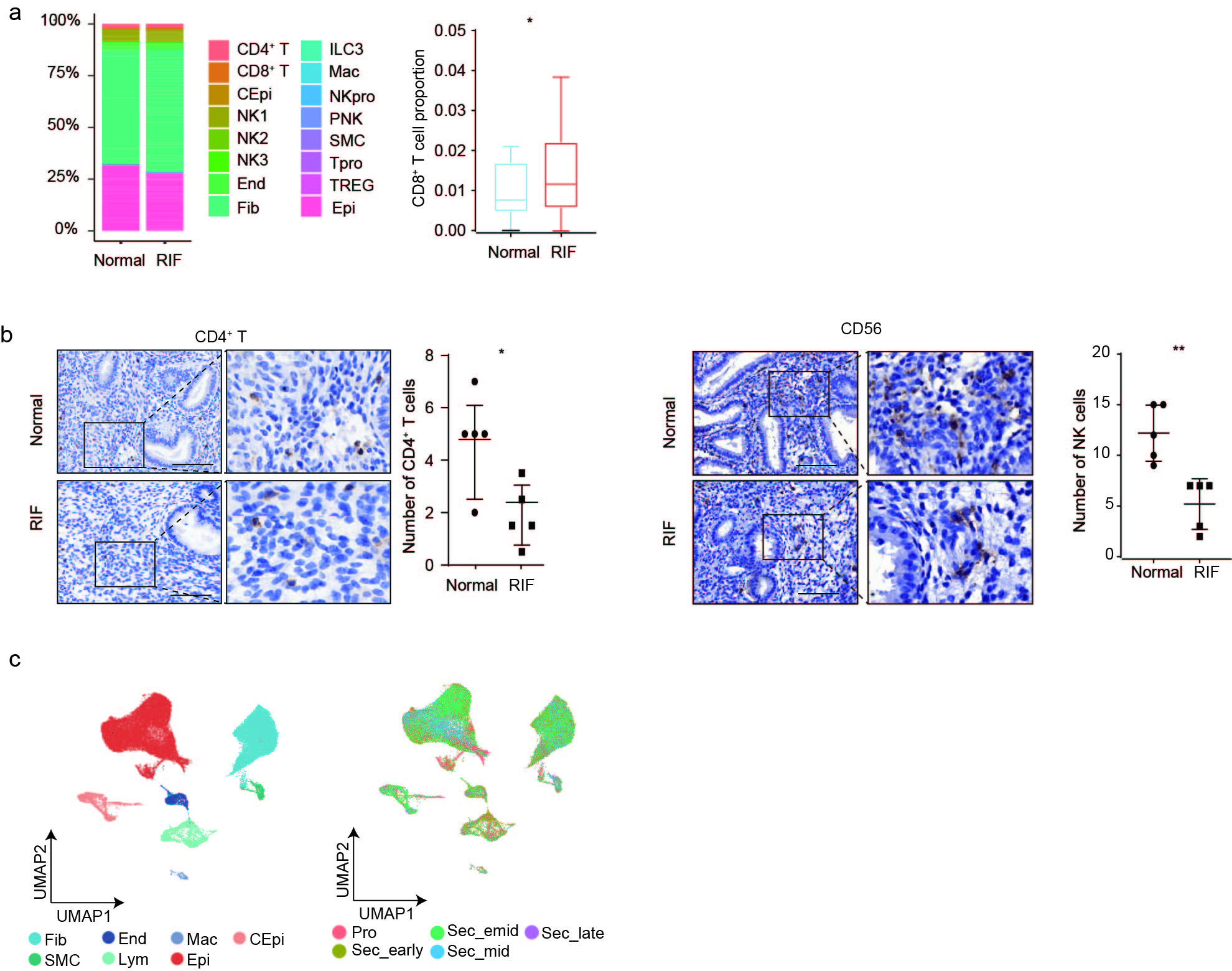
Supplementary Fig. S3 | Validation of the proportions of lymphocytes and CD8^+^ T-cells during the WOI and in patients with unexplained RIF. a** Left: Relative abundance of every type of endometrial cell type in the bulk sequence calculated by CIBERSORTx. Right: The proportion of CD8^+^ T-cells among the endometrial cells from the controls and patients with unexplained RIF calculated by CIBERSORTx. Data are presented as median ± IQR. *P* values were calculated by two-sided unpaired Mann–Whitney *U* test. *, *P* < 0.05. **b** 1. Left**:** Immunohistochemical staining for the expression of CD4 in the endometria of the controls and patients with unexplained RIF. Scale bar, 100 μm. Right: the count pf CD4^+^ T-cells per field in the endometria of the controls and patients with unexplained RIF. *P* values were determined by the Wilcoxon test. 2. Left**:** Immunohistochemical staining for the expression of CD56 in the endometria of the controls and patients with unexplained RIF. Scale bar, 100 μm. Right: the number of CD56^+^ cells per field in endometria from the controls and patients with unexplained RIF endometrium. Data are presented as median ± IQR. *P* values were calculated by two-tailed Student’s *t*-test. *, *P* < 0.05; **, *P* < 0.01. **c** Left: UMAP visualization of cells from the endometria of 10 controls across different phases of the menstrual cycle. Right: previously published UMAP visualization of cells from different phases of the menstrual cycle.


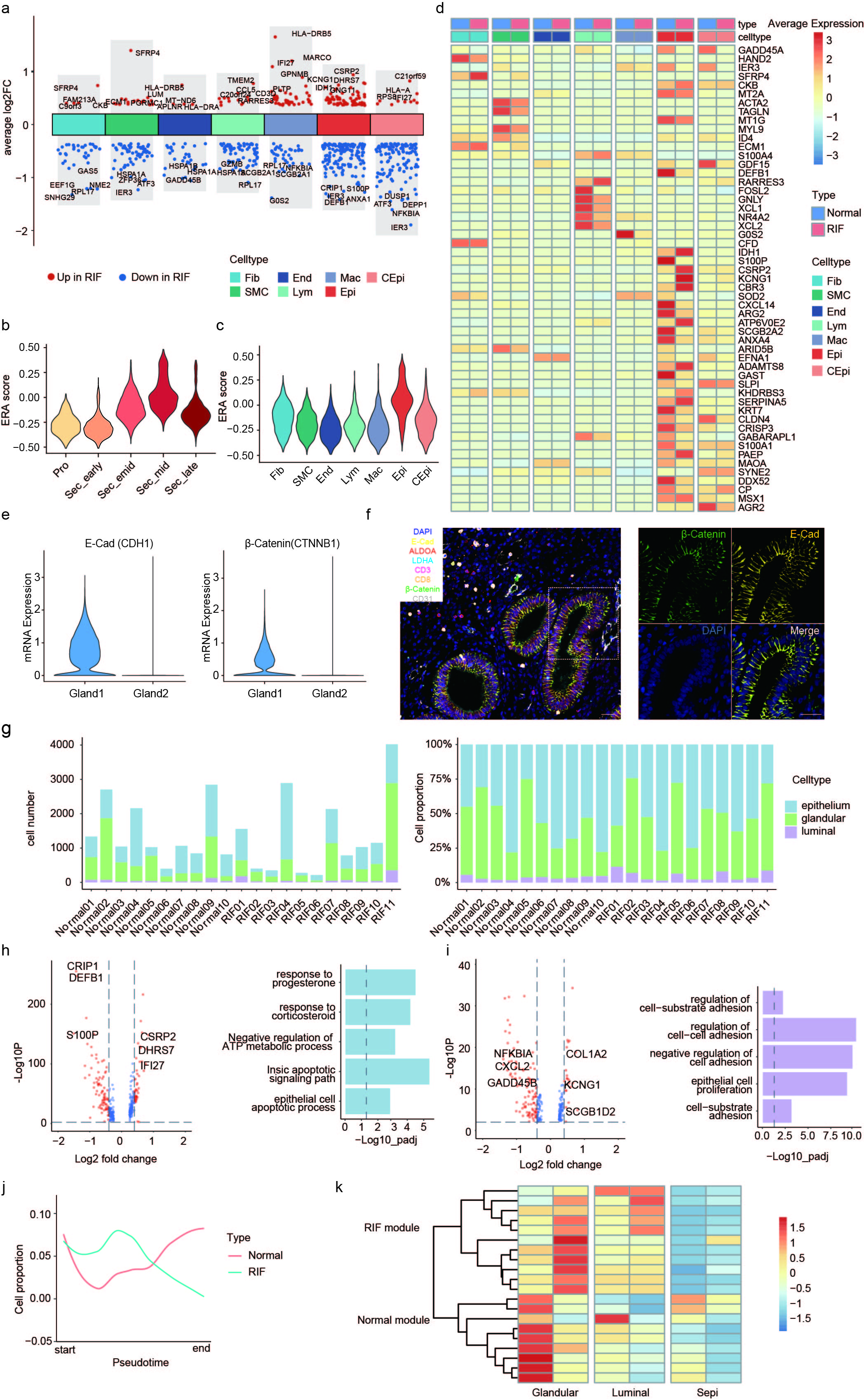


**Supplementary Fig. S4 | Differentiation log of the glandular epithelia of patients with unexplained RIF. a** Differential gene expression analysis showing up- and downregulated genes across all seven cell types. **b** Gene set enrichment analysis for the indicated signatures of different phases of the menstrual cycle (external data). **c** Gene set enrichment analysis for the indicated signatures of the seven cell types. **d** Heatmaps showing the expression of genes shared between the DEGs and the ERA for each endometrial cell type in the controls and patients with unexplained RIF. **e** Violin plot showing the expression of CDH1 and CTNNB1 genes in the subsets of gland epithelium. **f** Multiplex immunofluorescence imaging in human endometrium. **g** Cell number and relative proportion of epithelial subsets in each sample. **h, i** Left: Volcano plots representing differentially expressed genes within the support subset (f) and luminal subset (g) in the endometria of the controls and patients with unexplained RIF. Right: GO analysis of the DEGs of the support subset (f) and luminal subset (g) between the controls and patients with unexplained RIF. **j** Proportion of glandular epithelium in the endometria of the controls and patients with unexplained RIF endometrium along the pseudotime cell trajectory. **k** Heatmap showing the modules of the three epithelial subsets in the controls and patients with unexplained RIF. The modules consisted of a set of trajectory-related genes that varied between the controls and patients with unexplained RIF. The heatmap shows the aggregate expression of all genes in each module across all clusters.

**
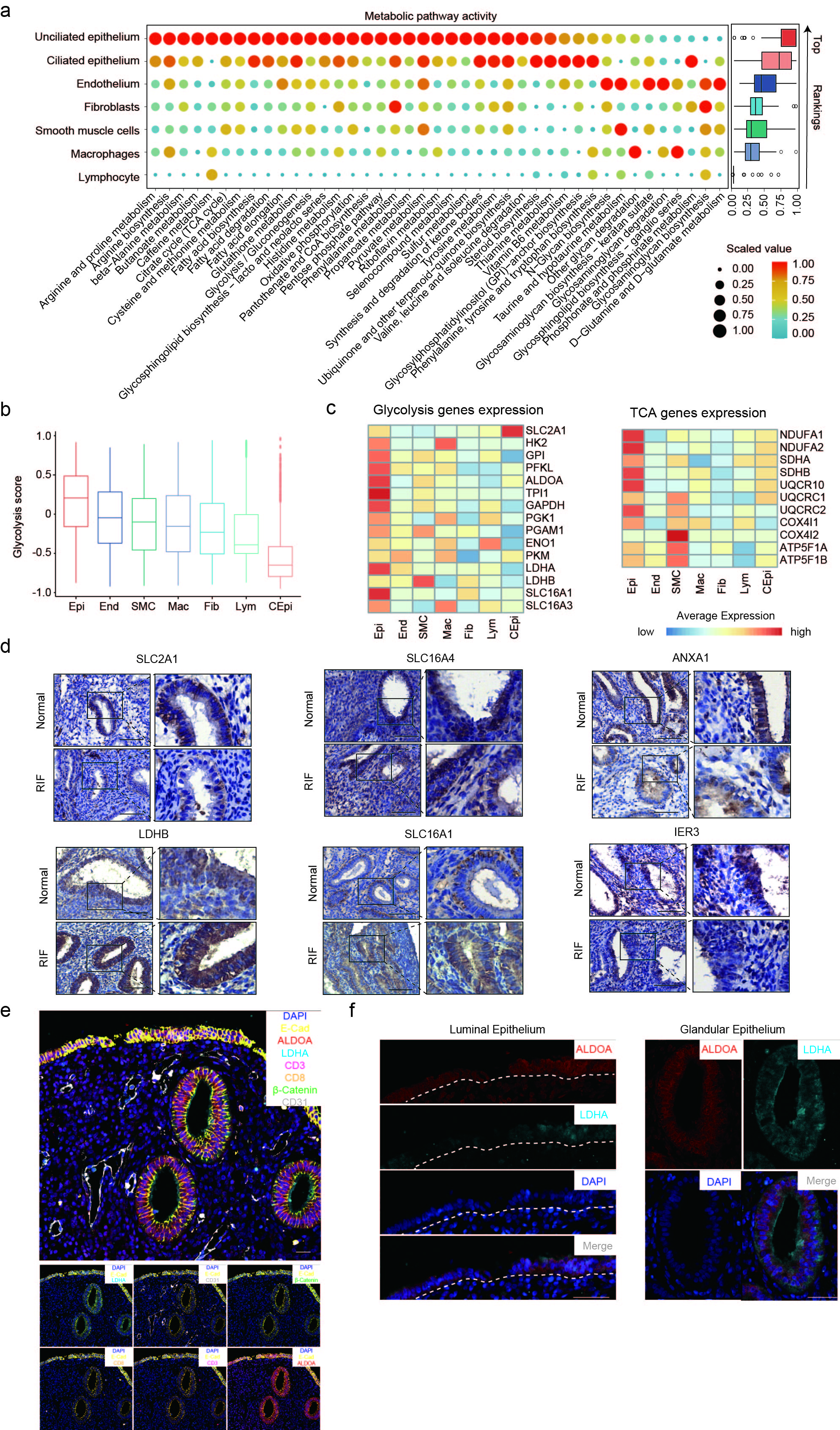
Supplementary Fig. S5 | Abnormal metabolism in the endometrial glandular epithelia of patients with unexplained RIF. a** Metabolic activity analysis of the endometrium revealed that the epithelium, especially in patients with unexplained RIF, had the highest metabolic score. The circle size and colour brightness both represent the scaled metabolic score. **b** Box plot showing the glycolysis levels among the 7 main cell types in the endometrium. **c** Expression of genes related to glycolysis and the TCA cycle among the 7 main types of endometrial cells. **d** IHC for the expression of SLC2A1, SLC16A4, ANXA1, LDHB, SLC16A1 and IER3 in human endometrial tissues from the controls and patients with unexplained RIF. Scale bar, 100 μm. **e,f** Representative regions of the luminal epithelium and glandular epithelium in control endometrial.

**
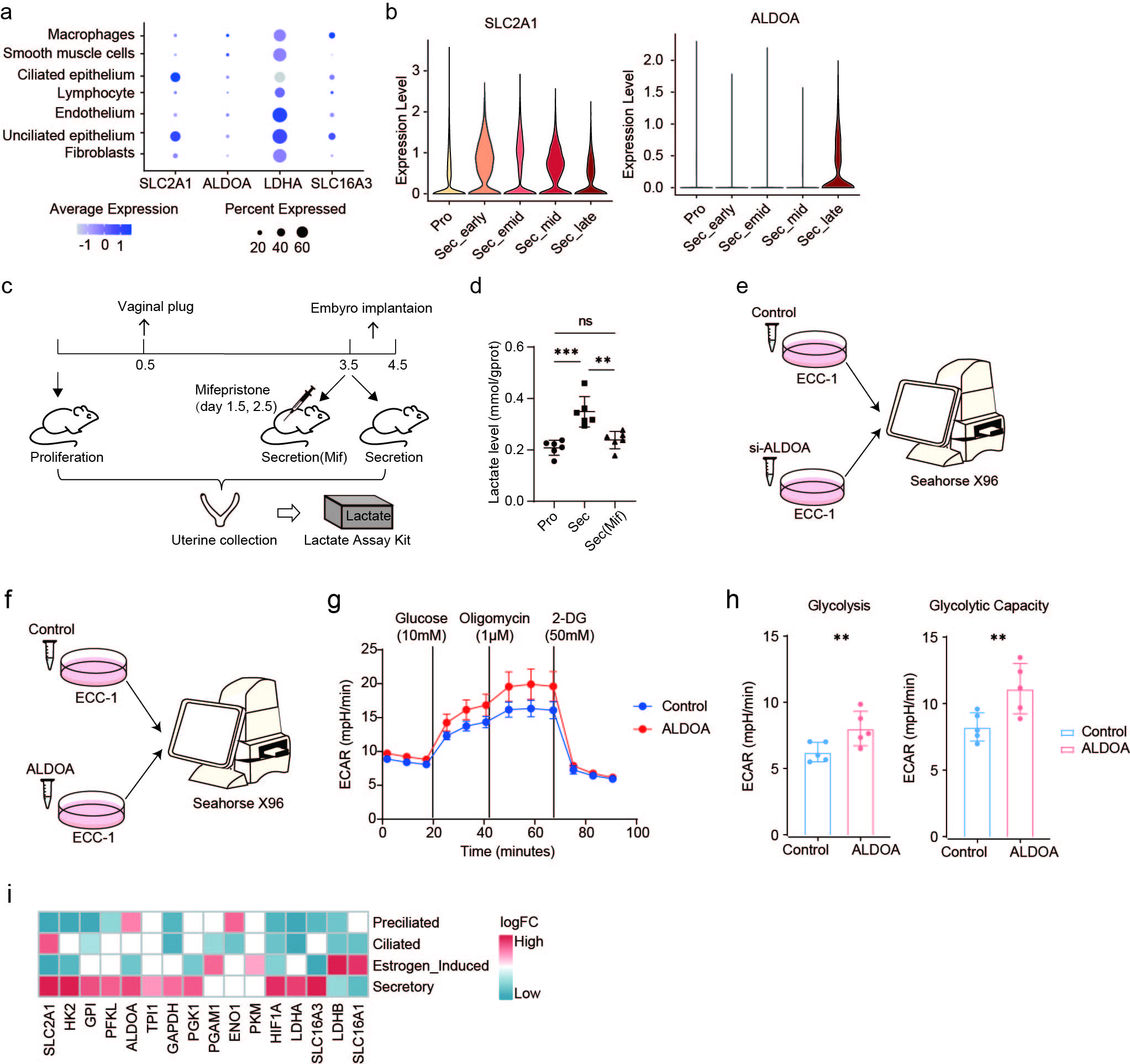
Supplementary Fig. S6 | Reduced lactate in the endometria of patients with unexplained RIF. a** Dot plot showing the expression of LA-related genes in the different cell types of the endometrium. **b** Dynamics of SLC2A1 and ALDOA expression in the endometria of the controls and patients with unexplained RIF throughout the menstrual cycle. **c** Schematic of the in vivo experiment. **d** Lactate level of mice uteri of three group (proliferation, secretion, and mifepristone). The data are presented as the mean ± s.d. and were analysed by two-tailed Student’s t-test (n=6 per group). **, P < 0.01; ***, P < 0.001. **e** and **f** Schematic of the experiment. **g, h** ECAR changes, glycolysis rates, and glycolysis capacities were measured. The data are presented as the mean ± s.d. and were analysed by two-tailed Student’s *t*-test. *, *P* < 0.05. **i** In vitro responses of endometrial organoids to ovarian hormones. Heatmaps showing glycolysis genes differentially expressed in ciliated and secretory lineages. Color is proportional to log-transformed fold change. Upon further stimulation with progesterone (label as secretory), glandular epithelium had higher expression of genes involved in glycolysis.

**
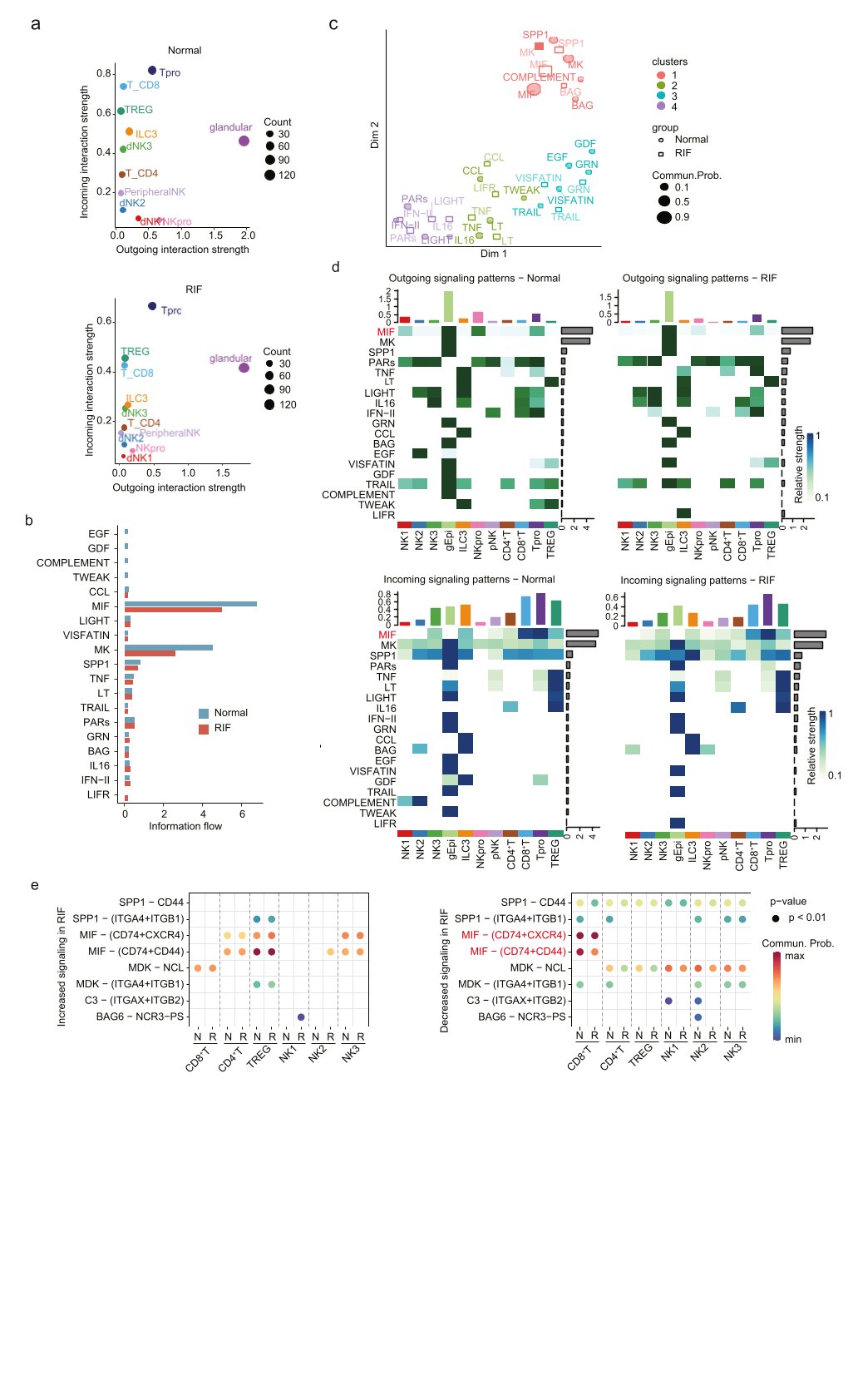
Supplementary Fig. S7 | Signalling from the glandular epithelium to lymphocytes. a** Projecting cell types onto a two-dimensional manifold according to their interaction strength similarity. (Top: controls; bottom: patients with unexplained RIF) **b** All significant signalling pathways were ranked based on their differences in information flow within the inferred networks between the controls and patients with unexplained RIF. The top signalling pathways coloured blue were more enriched in the controls, the middle ones coloured black were equally enriched in the controls and patients with unexplained RIF, and the bottom ones coloured red were more enriched in the patients with unexplained RIF. **c** Functional classification of pathways between the glandular epithelium and lymphocytes. Projecting signalling pathways onto a two-dimensional manifold according to their functional similarity. Each dot represents the communication network of one signalling pathway. The dot size is proportional to the overall communication probability. Different colours represent different groups of signalling pathways. **d** Outgoing (upper) and incoming (lower) signalling patterns in different cell types. The darker the colour is, the greater the relative strength of the signalling pattern in the cell type. Bar plot at the top: total relative strength of signalling patterns in each cell type. Bar plot on the right: total relative strength of each signalling pattern in all cell types. **e** Comparison of the significant ligand-receptor pairs between patients with unexplained RIF and the controls, which contribute to signalling from the glandular epithelium to lymphocytes (increased signalling in patients with unexplained RIF). The colour of the dots reflects the communication probability, and the dot size represents the computed p value. An empty space means that the communication probability is zero. p values were computed from a one-sided permutation test.


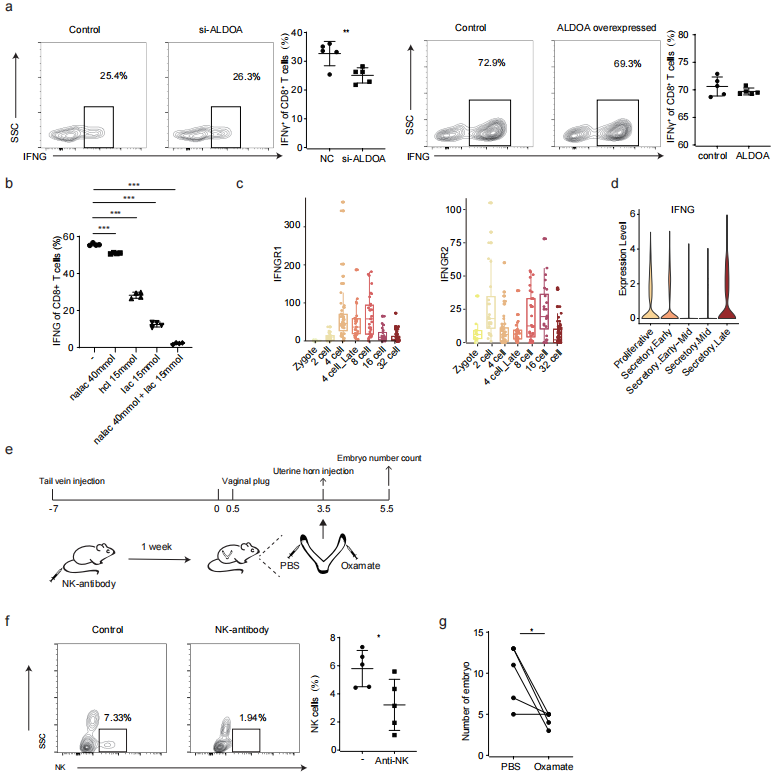


**Supplementary Fig. S8 | Insufficient lactate causes implantation failure by impairing immune balance. a** Flow cytometric analysis of IFNG^+^ cells among stimulated CD8^+^ T-cells after incubation in the supernatant of control (-) and si-ALDOA/ALDOA-overexpressing ECC-1 epithelia for 48 h. The data are presented as the mean ± s.d. and were analysed by two-tailed Student’s *t*-test. **, *P* < 0.01. **b** Flow cytometric analysis of IFNG^+^ cells among stimulated CD8^+^ T-cells after incubation in lactate (nalac), hydrochloric acid (hcl) or hcl for 48 h. The data are presented as the mean ± s.d. and were analysed by two-tailed Student’s *t*-test. ***, P < 0.001. **c** The expression of IFNG receptor at different embryo stages. **d** Dynamics of IFNG expression in the lymphocyte of normal endometria across the menstrual cycle. **e** Schematic of the experiment. **f** Flow cytometric analysis of IFNG^+^ cells among stimulated CD8^+^ T-cells after incubation in the supernatant of control (-) and si-ALDOA/ALDOA-overexpressing ECC-1 epithelia for 48 h. **g** Flow cytometric analysis of CD8^+^ T-cells in the peripheral blood on Day 5.5 of pregnancy. Left: control mice; right: mice subjected to anti-NK treatment. The data are presented as the mean ± s.d. and were analysed by two-tailed Student’s *t*-test. *, *P* < 0.01. **d** Number of embryo implantation sites in uteri injected with PBS or oxamate after anti-NK treatment. *P* values were calculated by two-sided paired Mann–Whitney *U* test (n=10 per group). **, *P* < 0.01.
